# Cardiovascular Events After Chimeric Antigen Receptor T-Cell Therapy for Advanced Hematologic Malignant Neoplasms: A Meta-Analysis

**DOI:** 10.1001/jamanetworkopen.2024.37222

**Published:** 2024-10-07

**Authors:** David Koeckerling, Rohin K. Reddy, Joseph Barker, Christian Eichhorn, Pip Divall, James P. Howard, Felix Korell, Michael Schmitt, Peter Dreger, Norbert Frey, Lorenz H. Lehmann

**Affiliations:** 1 Department of Cardiology, Angiology and Respiratory Medicine, University Hospital Heidelberg, Heidelberg, Germany; 2 German Centre for Cardiovascular Research (DZHK), partner site, Mannheim/Heidelberg, Germany; 3 National Heart and Lung Institute, Imperial College London, London, United Kingdom; 4 Nuffield Department of Population Health, University of Oxford, Oxford, United Kingdom; 5 Division of Acute Medicine, University Hospital Basel, Basel, Switzerland; 6 University Hospitals of Leicester National Health Service Trust, Leicester, United Kingdom; 7 Department of Hematology, Oncology & Rheumatology, University Hospital Heidelberg, Heidelberg, Germany; 8 German Cancer Research Centre (DKFZ), Heidelberg, Germany

## Abstract

**Importance:**

The frequency and clinical phenotypes of cardiotoxic events in chimeric antigen receptor (CAR) T-cell recipients remain poorly understood given that landmark approval trials typically exclude patients with high-risk cardiovascular profiles and data from nontrial settings are scarce.

**Objective:**

To summarize the prevalence of adverse cardiovascular events among adults receiving CAR T-cell therapies for advanced hematologic malignant neoplasms.

**Data Sources:**

MEDLINE, Embase, Cochrane Library, and Google Scholar were systematically searched from database inception until February 26, 2024.

**Study Selection:**

Observational studies were included if they comprised adult CAR T-cell recipients with advanced hematologic malignant neoplasms and if they systematically evaluated cardiovascular complications.

**Data Extraction and Synthesis:**

Extraction of prespecified parameters related to the patient population, study design, and clinical events was performed at the study level by 2 independent reviewers in accordance with the Meta-Analysis of Observational Studies in Epidemiology (MOOSE) reporting guideline. Meta-analysis of single proportions was conducted using random-effect models with Freeman-Tukey double arcsine transformations to calculate pooled prevalence estimates. Sensitivity analysis was performed using generalized linear mixed models with logit transformations.

**Main Outcomes and Measures:**

Ventricular and supraventricular arrhythmias, heart failure events, reduction in left ventricular ejection fraction, myocardial infarction, and cardiovascular and all-cause mortality.

**Results:**

Thirteen studies comprising 1528 CAR T-cell recipients (median [IQR] age, 61 [58.7-63.0] years; 1016 males [66%]; 80% patients with lymphoma) were included. The median (IQR) duration of follow-up was 487 (294-530) days. On random-effects meta-analysis, we observed a pooled prevalence of 0.66% (95% CI, 0.00%-2.28%) for ventricular arrhythmia, 7.79% (95% CI, 4.87%-11.27%) for supraventricular arrhythmia, 8.68% (95% CI, 2.26%-17.97%) for left ventricular dysfunction, 3.87% (95% CI, 1.77%-6.62%) for heart failure events, 0.62% (95% CI, 0.02%-1.74%) for myocardial infarction, and 0.63% (95% CI, 0.13%-1.38%) for cardiovascular death. The pooled prevalence of all-cause mortality was 30.01% (95% CI, 19.49%-41.68%). Sensitivity analyses generated similar findings.

**Conclusions and Relevance:**

This meta-analysis found a low prevalence of ventricular arrhythmia, myocardial infarction, and cardiovascular death among CAR T-cell recipients over a short-term to midterm follow-up. Left ventricular dysfunction and supraventricular arrhythmia were the most commonly reported cardiovascular complications, suggesting that cardiovascular surveillance strategies should focus on decreases in ejection fraction and supraventricular arrhythmia.

## Key Points

**Question:**

What is the prevalence of specific cardiovascular events in adults receiving chimeric antigen receptor (CAR) T-cell products for advanced hematologic malignant neoplasms?

**Findings:**

In this meta-analysis of 13 studies comprising 1528 CAR T-cell recipients, a low pooled prevalence of serious adverse cardiac events, such as ventricular arrhythmia, myocardial infarction, and cardiovascular death, was observed. The most commonly observed complications were left ventricular dysfunction and supraventricular arrhythmia.

**Meaning:**

Findings of this study suggest that cardiovascular surveillance strategies in CAR T-cell recipients should focus on decreases in ejection fraction and supraventricular arrhythmia.

## Teaser

This meta-analysis assesses the prevalence of ventricular and supraventricular arrhythmias, left ventricular dysfunction, heart failure, myocardial infarction, and death among patients who were treated with chimeric antigen receptor (CAR) T-cell products.

## Introduction

Chimeric antigen receptor (CAR) T-cell therapy has demonstrated extraordinary potency and promise in the treatment of select refractory or relapsed hematologic malignant neoplasms. CAR T cells are genetically engineered, autologous T lymphocytes designed to selectively target specific antigens and induce cellular apoptosis. CAR is a synthetic recombinant fusion protein composed of an extracellular single-chain fragment of a monoclonal antibody fused with a costimulatory molecule such as CD28 or CD137/4-1BB and the intracellular domain of the T-cell receptor, enabling the redirection of T lymphocytes to exert antitumor immune responses without the need for antigen presentation via major histocompatibility complexes. In view of unprecedented response rates achieved in patients with previously highly limited curative options, ongoing research efforts focus on the expansion of CAR T-cell therapy into solid tumors and nonmalignant pathologies, such as autoimmune disease, cardiac disease, and HIV.^1,2,3,4^

Despite successfully reshaping the oncological treatment landscape, CAR T-cell therapy has also been linked to serious generalized and organ-specific complications, such as the cytokine release syndrome (CRS), immune cell–associated neurotoxicity, and immune cell–associated hematotoxicity. However, the cardiotoxic profile of CAR T-cell therapies remains poorly understood due to underrepresentation of patients with preexisting cardiovascular comorbidities in landmark clinical trials and the paucity of data generated in nontrial settings.^5,6,7,8^ As CAR T-cell therapies progressively transition into the standard of care for various malignant neoplasms and the spectrum of eligible patients continues to expand, it is pivotal to define the cardiotoxic potential of CAR T-cell therapy for optimal integration of cardio-oncology care. Accordingly, this meta-analysis aimed to summarize the prevalence of adverse cardiovascular events, including ventricular and supraventricular arrhythmias, heart failure events, left ventricular dysfunction, myocardial infarction, and cardiovascular death, among adults receiving CAR T-cell therapies for advanced hematologic malignant neoplasms.

## Methods

This meta-analysis followed the Meta-Analysis of Observational Studies in Epidemiology (MOOSE) reporting guideline.^9^ It was registered on PROSPERO (CRD42024496470).

### Search Strategy

The electronic databases MEDLINE, Embase, Cochrane Library, and Google Scholar were systematically searched by a clinical librarian (P.D.) from database inception through February 26, 2024. No language restrictions were applied. The search syntax was designed using a combination of key words and MeSH (Medical Subject Headings) around the concepts of CAR T-cell therapy and cardiovascular events; the full search strategy is provided in eAppendix 1 in Supplement 1. Conference abstracts and ongoing clinical trial records were excluded. References of included studies were manually searched to identify additional eligible studies. After duplicate records were deleted, abstract screening was conducted in a blinded manner by 2 independent reviewers (D.K., J.B.) using systematic review software (Rayyan Systems; Rayyan).

### Study Selection

Observational studies were eligible for inclusion if the following criteria were met. First, the study population consisted of adults with refractory or relapsed hematologic malignant neoplasms who were receiving at least 1 of the following commercially available CAR T-cell therapies: axicabtagene ciloleucel (Yescarta; Kite Pharma Inc), tisagenlecleucel (Kymriah; Novartis Pharmaceuticals Corp), brexucabtagene autoleucel (Tecartus; Kite Pharma Inc), lisocabtagene maraleucel (Breyanzi; Bristol Myers Squibb), or idecabtagene vicleucel (Abecma; Bristol Myers Squibb). Second, 1 or more of the following outcomes were reported: cardiovascular death, ventricular arrhythmia, supraventricular arrhythmia, heart failure events, myocardial infarction, and/or reduction in left ventricular ejection fraction (LVEF). We excluded clinical trials conducted for approval purposes and focused on oncological effectiveness end points given that patients with cardiovascular comorbidities are predominantly excluded in such settings and systematic assessment of cardiotoxic effects is not performed. In addition, we excluded studies assessing cardiac events based on the US Food and Drug Administration Adverse Event Reporting System since this pharmacovigilance database consists of voluntarily reported adverse events and does not include patients without complications.^10^

Full-text review, data extraction, and risk-of-bias assessment were undertaken by 2 independent investigators (D.K., J.B.), and interreviewer discrepancies were resolved by consensus or by discussion with senior investigators. In case of missing data, senior authors of the study were contacted to request additional information. Risk-of-bias assessment was performed at the study level using the Joanna Briggs Institute critical appraisal checklist for prevalence data, a quality assessment tool specifically developed and formally evaluated for use in meta-analyses of prevalence data.^11,12^

### Outcomes

The outcomes of interest were ventricular arrhythmia, supraventricular arrhythmia, reduction in LVEF, heart failure events, myocardial infarction, and cardiovascular and all-cause mortality. The composite end point of major adverse cardiovascular events was not collected due to substantial interstudy heterogeneity in the definition of this outcome. Events were considered to be heart failure related when at least 2 of the following criteria were met: symptoms (exertional dyspnea, decreased exercise tolerance, orthopnea, or volume overload), signs (peripheral edema, bibasal pulmonary crackles, increased jugular venous pressure, or weight gain related to fluid retention), or laboratory or imaging parameters (radiological evidence of pulmonary congestion, elevated N-terminal pro-B-type natriuretic peptide, or vena cava inferior over 20 mm in diameter without respiratory fluctuation) consistent with heart failure as well as initiation or escalation of heart failure treatment (diuretics and inotropes or mechanical support). Supraventricular arrhythmias were defined as supraventricular tachycardia, atrial fibrillation, or flutter. Reduction in LVEF was defined as a decrease of more than 10% from baseline to a value of less than 50%. Myocardial infarction was defined as a combination of troponin elevation above the 99th percentile of the normal range, ischemic electrocardiographic changes, and/or symptoms of myocardial ischemia. Cardiovascular death was defined as mortality consequent to heart failure, cardiogenic shock, myocardial infarction, or arrhythmia.

### Statistical Analysis

Meta-analysis of single proportions was conducted using inverse-variance random-effect models to calculate pooled prevalence estimates. The 95% CIs for prevalence were calculated using the exact binomial (Clopper-Pearson) method. In practice, proportional data commonly violate the assumption of normality due to their skewness. Additionally, when a proportion is small or large (ie, close to 0 or 1), variance tends toward 0; consequently, studies may receive inappropriately large weights within the meta-analysis model. Therefore, the Freeman-Tukey double arcsine transformation was applied to mitigate these problems.^13^ The final pooled result and 95% CIs were then back-transformed and expressed as percentages for ease of interpretation. The Freeman-Tukey transformation displays increased variance stability compared with the logit transformation, making it the preferred method in meta-analysis of proportions,^14,15^ although it has raised some concerns when aggregating studies with different sample sizes.^16^

Although the range of study populations was reasonably narrow, sensitivity analyses using generalized linear mixed (ie, random intercept logistic regression) models with the logit transformation were conducted to identify the robustness of results across analytic methods. Subgroup analysis was performed for prospective and retrospective study designs. The between-study variance, τ^2^, was estimated using the restricted maximum likelihood estimator in the Freeman-Tukey double arcsine transformation analyses and the maximum likelihood estimator in the generalized linear mixed models. Statistical heterogeneity was assessed using the *I*^2^ statistic. Potential modifiers of prevalence estimates (age and type of underlying malignant neoplasm) were explored using random-effects meta-regression for end points, where at least 10 studies were available for quantitative synthesis. Use of conventional measures for publication bias assessment, such as funnel plots and the Egger test, is generally not recommended in meta-analyses of prevalence because these tests were developed in the context of comparative data and may generate erroneous results when applied to proportional data.^14,15^ Instead, small-study effects, including publication bias, were assessed using Doi plots and Luis Furuya-Kanamori indices, which have shown improved sensitivity and specificity over traditional measures and retain their interpretability when applied to proportional data.^17^

Two-sided *P* < .05 indicated statistical significance. All analyses were performed using the meta package in R, version 4.3.1 (R Project for Statistical Computing).

## Results

After screening of 771 abstracts and full-text review of 86 reports, we included in the final analysis 13 studies^18,19,20,21,22,23,24,25,26,27,28,29,30^ comprising 1528 patients (median [IQR] age, 61.0 [58.7-63.0] years; 1016 males [66%], 512 females [34%]). The study selection process is provided in eFigure 1 in Supplement 1. The majority of included studies were conducted in the US (11 [84%]^18,19,20,21,23,24,25,26,27,28,30^) and had a retrospective (10 [77%]^18,19,20,23,24,26,27,28,29,30^) and single-center (10 [77%]^19,21,22,23,24,25,26,28,29,30^) design. The median (IQR) duration of follow-up was 487 (294-530) days (Table).

**Table.** Study and Patient Characteristics

Characteristic	Median (IQR)
**Study characteristics**	
Cohort size, No. (%)	
<100	5 (38)
≥100	8 (62)
Design, No. (%)	
Prospective	3 (23)
Retrospective	10 (77)
Single center	10 (77)
Multicenter	3 (23)
Origin, No. (%)	
US	11 (85)
Europe	1 (8)
Asia	1 (8)
Duration of follow-up, d	487 (294-530)
**Patient characteristics**	
Age, y	61.0 (58.7-63.0)
Sex, No. (%)	
Male	1016 (66)
Female	512 (34)
BMI	27.3 (27.0-28.4)
Diabetes, %	12.5 (9.8-17.0)
Hypertension, %	37.5 (34.7-43.0)
Hypercholesterolemia, %	32.0 (22.8-38.5)
Smoking history, %	35.0 (21.9-40.4)
Atrial fibrillation, %	12.0 (8.4-13.0)
Heart failure, %	5.5 (3.7-7.8)
Coronary artery disease, %	8.9 (7.1-10.2)
LVEF, %	59.8 (58.3-61.0)
Underlying malignant neoplasm, %	
Leukemia	7.9 (0.8-20.8)
Lymphoma	80.0 (30.0-98.0)
Myeloma	0.0 (0.0-8.5)
CAR T-cell product, %	
Axicabtagene ciloleucel (Yescarta)	50.0 (39.0-61.1)
Tisagenlecleucel (Kymriah)	12.0 (6.0-16.8)
Brexucabtagene autoleucel (Tecartus)	0.0 (0.0-4.0)
Previous anthracycline therapy, %	85.0 (80.0-87.1)
Previous radiotherapy, %	17.5 (10.5-26.8)
Previous stem cell transplant, %	27.0 (24.3-40.0)
CRS, %	
Overall	75.0 (60.0-81.7)
Grade ≥2^a^	31.0 (17.0-54.0)
Tocilizumab use	49.6 (41.4-55.4)

Abbreviations: BMI, body mass index (calculated as weight in kilograms divided by height in meters squared); CAR, chimeric antigen receptor; CRS, cytokine release syndrome; LVEF, left ventricular ejection fraction.

^a^ Grades 1 to 4, with higher grades indicating increased severity.

Enrolled patients received CAR T-cell therapy predominantly for lymphoma (median [IQR], 80.0% [30.0%-98.0%]), with considerable proportions of patients having previously undergone anthracycline therapy (median [IQR], 85.0% [80.0%-87.1%]), radiotherapy (median [IQR], 17.5% [10.5%-26.8%]), or stem cell transplant (median [IQR], 27.0% [24.3%-40.0%]). Cardiovascular risk factors were common among the study population, with a median (IQR) of 12.5% (9.8%-17.0%) having diabetes, 37.5% (34.7%-43.0%) having hypertension, 32.0% (22.8%-38.5%) having hypercholesterolemia, and 35.0% (21.9%-40.4%) reporting a smoking history. Study and patient characteristics are summarized in the Table, while methodological aspects are described in eTable 1 in Supplement 1.

Risk-of-bias assessment of included studies is depicted in eAppendix 2 in Supplement 1. Higher risks of bias were observed in categories pertaining to the identification and measurement of outcomes, reflecting the predominantly retrospective study design, reliance on electronic health record review, and lack of systematic pretreatment and posttreatment cardiac testing.

### Ventricular and Supraventricular Arrhythmias

**Figure 1.**
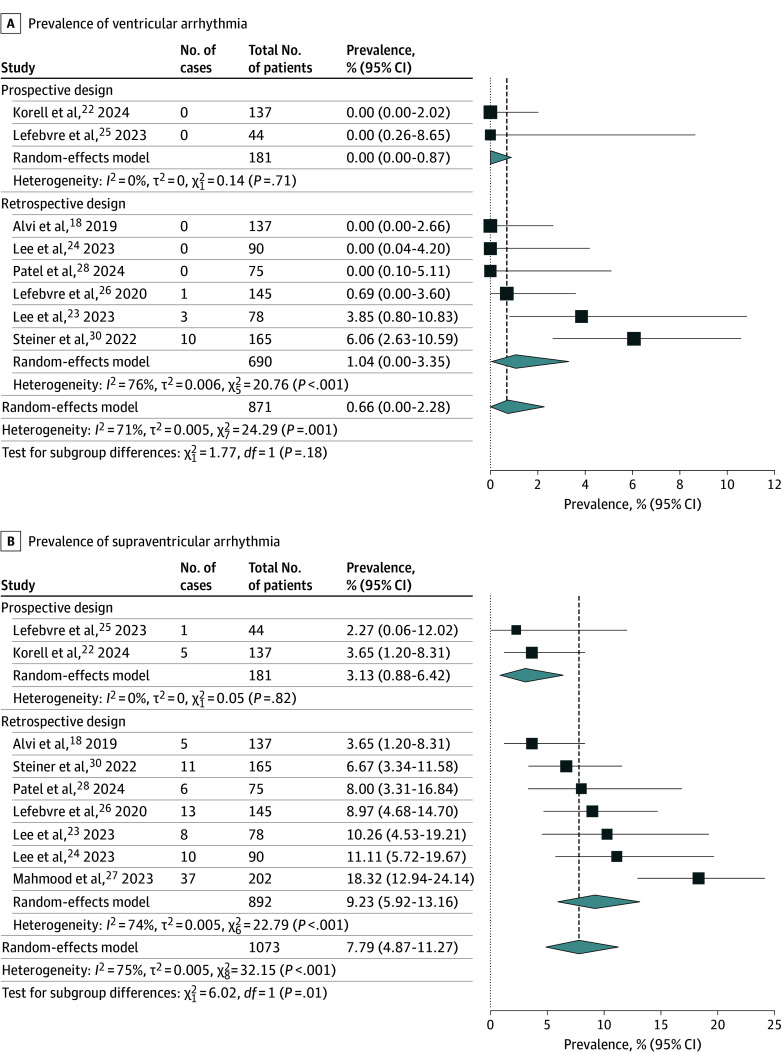
Pooled Prevalence of Ventricular Arrhythmia and Supraventricular Arrhythmia Inverse-variance random-effects models with Freeman-Tukey double arcsine transformation were used. Error bars represent 95% CIs. Diamonds indicate the pooled prevalence estimate and its associated 95% CI. Size of the squares represents weight.

The occurrence of ventricular arrhythmia in patients receiving CAR T-cell therapy was assessed in 8 studies^18,22,23,24,25,26,28,30^ comprising 871 patients (Figure 1A). Overall, 14 cases of ventricular arrhythmia were recorded over a median (IQR) follow-up duration of 487 (294-493) days. On random-effects meta-analysis using the Freeman-Tukey double arcsine transformation, the pooled prevalence of ventricular arrhythmia was 0.66% (95% CI, 0.00%-2.28%; *I*^2^ = 71%). On subgroup analysis of prospective studies (n = 181), the pooled prevalence of ventricular arrhythmia was 0.00% (95% CI, 0.00%-0.87%).

The occurrence of supraventricular arrhythmia was assessed in 9 studies^18,22,23,24,25,26,27,28,30^ comprising 1073 patients (Figure 1B). Overall, 96 cases of supraventricular arrhythmia were recorded over a median (IQR) follow-up duration of 418 (308-492) days. On random-effects meta-analysis using the Freeman-Tukey double arcsine transformation, the pooled prevalence of supraventricular arrhythmia was 7.79% (95% CI, 4.87%-11.27%; *I*^2^ = 75%). On subgroup analysis of prospective studies (n = 181), the pooled prevalence of supraventricular arrhythmia was 3.13% (95% CI, 0.88%-6.42%).

### Left Ventricular Dysfunction and Heart Failure Events

**Figure 2.**
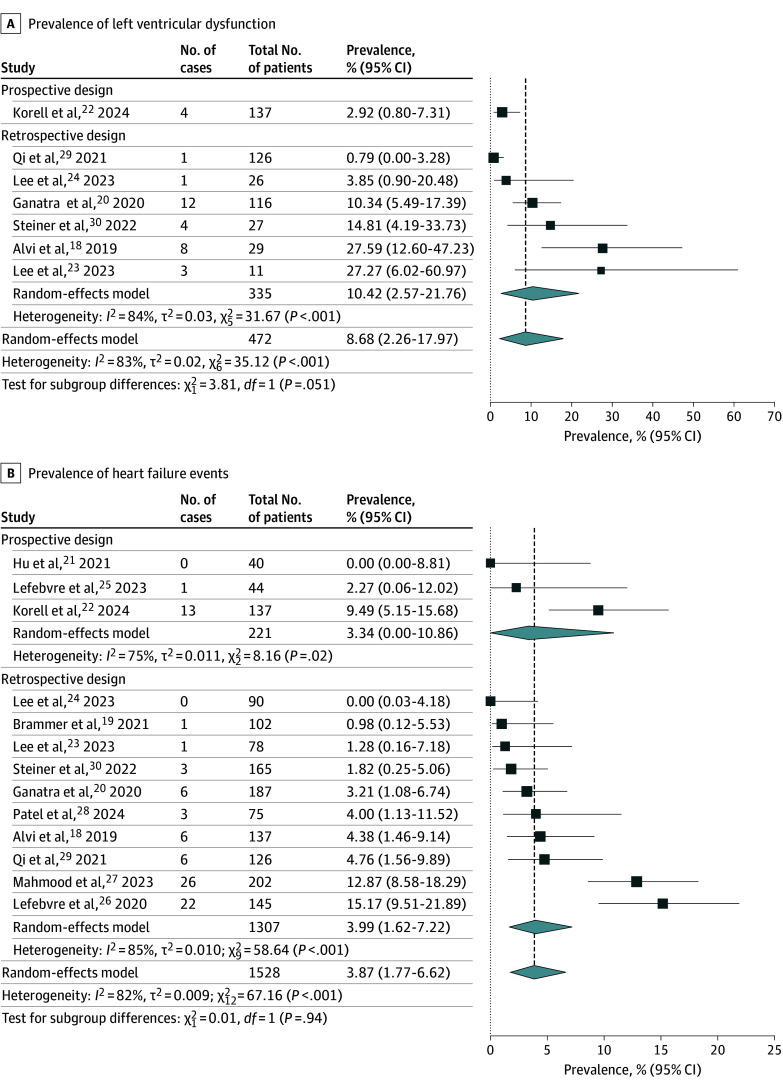
Pooled Prevalence of Reduction in Left Ventricular Dysfunction and Heart Failure Events Inverse-variance random effect models with Freeman-Tukey double arcsine transformation were used. Error bars represent 95% CIs. Diamonds indicate the pooled prevalence estimate and its associated 95% CI. Size of the squares represents weight.

Left ventricular dysfunction was reported in 7 studies^18,20,22,23,24,29,30^ including 472 patients (Figure 2A). There were 33 reported cases of left ventricular dysfunction over a median (IQR) follow-up of 285 (249-344) days. On random-effects meta-analysis using the Freeman-Tukey double arcsine transformation, the pooled prevalence of left ventricular dysfunction was 8.68% (95% CI, 2.26%-17.97%; *I*^2^ = 83%). Subgroup analysis of prospective studies was not feasible due to paucity of data.

All 13 studies^18,19,20,21,22,23,24,25,26,27,28,29,30^ comprising 1528 patients evaluated the occurrence of heart failure events (Figure 2B). Overall, 76 heart failure events were reported over a median (IQR) follow-up duration of 487 (294-530) days, resulting in a pooled prevalence of 3.87% (95% CI, 1.77%-6.62%; *I*^2^ = 82%). On subgroup analysis of prospective studies (n = 221), the pooled prevalence of heart failure events was 3.34% (95% CI, 0.00%-10.86%).

### Myocardial Infarction and Cardiovascular and All-Cause Mortality

**Figure 3.**
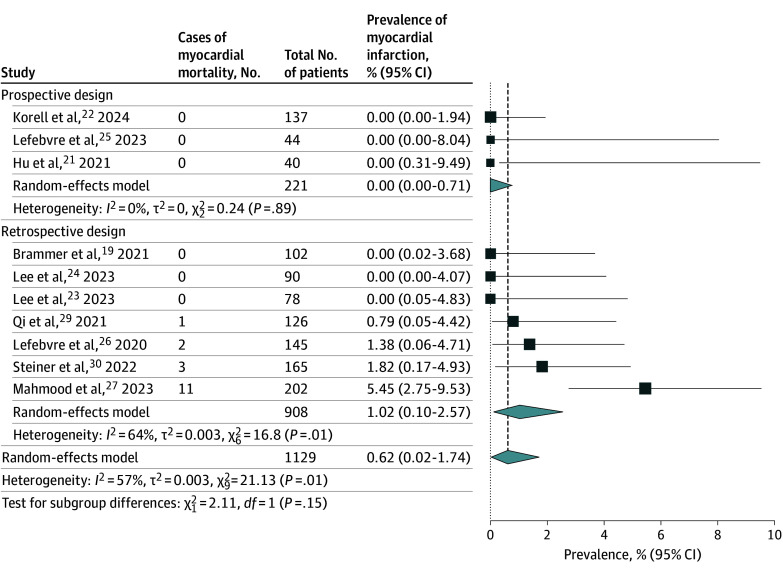
Pooled Prevalence of Myocardial Infarction Inverse-variance random effect models with Freeman-Tukey double arcsine transformation were used. Error bars represent 95% CIs. Diamonds indicate the pooled prevalence estimate and its associated 95% CI. Size of the squares represents weight.

The occurrence of myocardial infarction was assessed in 10 studies^19,21,22,23,24,25,26,27,29,30^ comprising 1129 patients (Figure 3). Overall, 17 cases of myocardial infarction were reported over a median (IQR) follow-up duration of 493 (418-565) days. On random-effects meta-analysis using the Freeman-Tukey double arcsine transformation, the pooled prevalence of myocardial infarction was 0.62% (95% CI, 0.02%-1.74%; *I*^2^ = 57%). On subgroup analysis of prospective studies (n = 221), the pooled prevalence of myocardial infarction was 0.00% (95% CI, 0.00%-0.71%).

**Figure 4.**
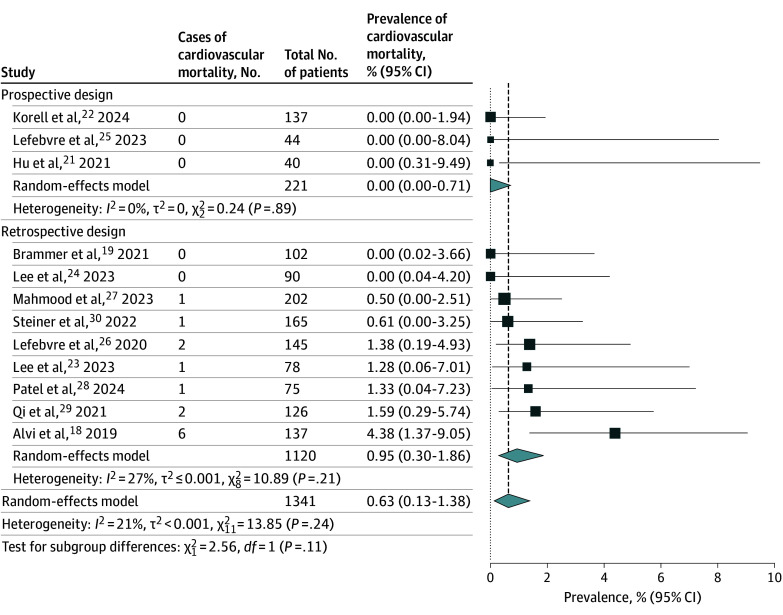
Pooled Prevalence of Cardiovascular Death Inverse-variance random effect models with Freeman-Tukey double arcsine transformation were used. Error bars represent 95% CIs. Diamonds indicate the pooled prevalence estimate and its associated 95% CI. Size of the squares represents weight.

Twelve studies^18,19,21,22,23,24,25,26,27,28,29,30^ comprising 1341 patients (Figure 4) reported 14 cases of cardiovascular mortality over a median (IQR) follow-up duration of 490 (335-547) days. On random-effects meta-analysis using the Freeman-Tukey double arcsine transformation, the pooled prevalence of cardiovascular mortality was 0.63% (95% CI, 0.13%-1.38%; *I*^2^ = 21%). On subgroup analysis of prospective studies (n = 221), the pooled prevalence of cardiovascular mortality was 0.00% (95% CI, 0.00%-0.71%).

The occurrence of all-cause mortality in patients receiving CAR T-cell therapy was assessed in 7 studies^21,22,23,24,25,26,27^ comprising 736 patients (eFigure 2 in Supplement 1). Overall, 262 patients died over a median (IQR) follow-up duration of 487 (349-599) days. On random-effects meta-analysis using the Freeman-Tukey double arcsine transformation, the pooled prevalence of all-cause mortality was 30.01% (95% CI, 19.49%-41.68%; *I*^2^ = 92%).

### Sensitivity Analysis and Meta-Regression

Sensitivity analysis using generalized linear mixed models with logit transformation methods yielded results consistent with those in the primary analyses (eFigures 3-9 in Supplement 1). On sensitivity analysis, we observed a pooled prevalence of 0.35% (95% CI, 0.03%-3.57%) for ventricular arrhythmia, 7.55% (95% CI, 5.07%-11.11%) for supraventricular arrhythmia, 7.59% (95% CI, 3.05%-17.62%) for left ventricular dysfunction, 3.40% (95% CI, 1.80%-6.33%) for heart failure events, 0.55% (95% CI, 0.12%-2.39%) for myocardial infarction, and 0.78% (95% CI, 0.33%-1.83%) for cardiovascular death. Random-effects meta-regression evaluating patient age and type of underlying malignant neoplasm (proportion of patients with lymphoma) as potential sources of heterogeneity were feasible for heart failure events (n = 76), myocardial infarctions (n = 17), and cardiovascular deaths (n = 14) (eTable 2 in Supplement 1). Prevalence estimates for heart failure events were modified by patient age (regression coefficient, –0.02; *P* = .01), while no significant modifiers were observed for other end points.

## Discussion

This proportional meta-analysis summarized the prevalence of adverse cardiovascular events in adults treated with CAR T-cell therapy for advanced hematologic malignant neoplasms. Cell-based antitumor therapies may play multifaceted roles in the cardiovascular system through direct (T-cell–mediated) and indirect (cytokine-mediated) mechanisms. Evidence demonstrating direct T cell–mediated cardiotoxic effects remains sparse,^31^ yet it has been hypothesized that cross-reactivity between CAR T cells and normal myocardial tissue may precede off-target, antimyocardial T-cell reactivity. Indirect cardiotoxic effects are believed to occur as an epiphenomenon of the CRS, as the interaction of activated CAR T cells with the tumor microenvironment precipitates a cascade of systemic proinflammatory immune responses with the potential to induce cytokine-mediated myocardial dysfunction (via interleukin [IL] 6 and tumor necrosis factor secretion), microvascular obstruction, distributive shock hemodynamics, systolic and diastolic left ventricular impairment, and global myocardial ischemia.^1,32^ The frequency and clinical phenotype of such cardiotoxic events in CAR T-cell recipients and the consequent need for cardio-oncological surveillance programs remain poorly defined since landmark clinical trials typically omitted patients with cardiovascular high-risk profiles and did not systematically evaluate specific cardiovascular complications.

In the present meta-analysis of 13 studies,^18,19,20,21,22,23,24,25,26,27,28,29,30^ we observed a low prevalence of serious adverse cardiac events in CAR T-cell recipients, such as ventricular arrhythmia (0.66%; 95% CI, 0.00%-2.28%), myocardial infarction (0.62%; 95% CI, 0.02%-1.74%), and cardiovascular death (0.63%; 95% CI, 0.13%-1.38%). The most commonly observed cardiac events were left ventricular dysfunction (8.68%; 95% CI, 2.26%-17.97%) and supraventricular arrhythmia (7.79%; 95% CI, 4.87%-11.27%). The findings remained consistent across different analytical approaches. Given the cardiovascular risk factor profile of included patients, the extensive previous exposure to anthracycline therapy and radiotherapy, the high incidence of CRS, and the substantial all-cause mortality rate across studies (30.01%; 95% CI, 19.49%-41.68%), the cardiotoxic potential of CAR T-cell therapies appears relatively modest in this context. In comparison to retrospective studies, prospectively generated data revealed even lower proportions of serious adverse cardiovascular events, with a pooled prevalence of 0% for ventricular arrhythmia, myocardial infarction, and cardiovascular death.^21,22,25^ The lower prevalence of cardiac complications observed in prospective settings may be explained by several aspects. First, prospective studies were generally conducted more recently, when increased clinical and scientific experience of CAR T-cell use likely were associated with earlier identification and more aggressive treatment of CRS using the IL-6 receptor antagonist tocilizumab. Given the close association between CRS and cardiac complications, the discrepancy in prevalence estimates may be partly attributable to a lower severity of CRS in prospectively designed studies. Second, retrospective studies remain at disproportional risk for selection, misclassification, and publication biases, which may have resulted in the overestimation of cardiac events.

While left ventricular dysfunction was the most commonly observed cardiac complication, this finding requires cautious interpretation since the retrospective studies did not incorporate systematic serial echocardiographic evaluation. Furthermore, information on the potential for recovery of ventricular function was limited. In the 2 prospective studies^22,25^ that incorporated serial echocardiographic assessment in their study design, significant reductions in LVEF were rare. In the largest prospective study to date, reductions in LVEF were reported in 4 of 137 patients (3%).^22^ In a second prospective study, no significant changes in LVEF were observed between study visits, yet the investigators noted temporary and modest decreases in global longitudinal strain.^25^

In the context of broad previous exposures to antitumor therapies with known cardiotoxic potential, such as anthracyclines (85.0% of participants) and mediastinal radiotherapy (17.5% of participants), the single-arm nature of available data made it difficult to ascertain whether CAR T-cell therapies themselves directly played a role in the cardiotoxic burden. In comparison, a meta-analysis of more than 22 000 patients with cancer who were treated with anthracyclines found that cardiovascular events occurred in 10.6% (95% CI, 3.5%-17.6%), cardiac death in 0.4% (95% CI, 0.0%-0.9%), and all-cause death in 31.9% (95% CI, 3.7%-60.2%) of patients, which are comparable to the prevalence estimates of the present study, albeit over a median follow-up of 9 years.^33^ Conversely, the close temporal association between CAR T-cell infusion and occurrence of cardiac events supports a potentially causal relationship. For example, in the studies by Lefebvre et al^26^ and Mahmood et al,^27^ patients experienced cardiac events at a median (IQR) of 11 (6-151) days and 12 (7-99) days, respectively, after CAR T-cell infusion.

Bearing in mind the limitations of available data, the findings from this meta-analysis suggest that CAR T-cell therapies generally display relatively low cardiotoxic potential in adult populations with advanced hematologic malignant neoplasms over short-term or midterm follow-up duration. Previous meta-analyses have reported a higher prevalence of adverse cardiac events, such as arrhythmia, myocardial infarction, and cardiovascular death.^34,35^ However, these studies did not apply recommended transformation methods to proportional data,^34,35^ possibly resulting in flawed estimation of prevalence.^14,15^ In addition, studies involving pediatric populations, pharmacovigilance studies, randomized clinical trials, and conference abstracts were pooled, generating a highly heterogeneous patient and study collective and reducing the generalizability of results to broader clinical settings. Moreover, prior meta-analyses have pooled the composite end point of adverse cardiovascular events despite substantial interstudy discrepancies in end-point composition and have not differentiated between supraventricular and ventricular arrhythmias, thereby further complicating the interpretation of findings.

While the cohort of participants included in this analysis represents the current state of dissemination and application of CAR T-cell therapies, patients presently eligible for CAR T-cell products, given their cost and novelty, remain a highly select and high-risk oncological collective with a diverse range of exposures to previous antitumor therapies with cardiotoxic potential. Expanding use, newly emerging indications, and decreasing costs of CAR T-cell therapies may generate future patient cohorts who differ substantially from the present patient cohort and in terms of their propensity for developing cardiovascular complications. Therefore, caution is advised when applying the findings of the present analysis to future CAR T-cell recipients. In addition, larger future cohorts with longer follow-up duration may enable the documentation of rare and serious cardiac complications, such as myocarditis, which may not have been reliably captured and reported in the currently available evidence base.

### Limitations

The present analysis is limited by the quality of the included studies. Most included studies had a retrospective, single-center design and consequently features small sample sizes, lacked systematic pretreatment workup and consistent posttreatment surveillance (serial echocardiography, cardiac biomarkers, and electrocardiographic monitoring), and posed risks for selection and misclassification biases. The vast majority of included studies were conducted in the US, impeding the generalizability of the findings to global patient cohorts. Follow-up of available studies was generally limited to the short-term and midterm duration; hence, the findings from the present analysis cannot be extrapolated toward long-term cardiovascular sequelae and cardiotoxic potential of CAR T-cell therapy. There was methodological and clinical interstudy heterogeneity regarding underlying hematologic malignant neoplasms, use of specific CAR T-cell products, and outcome definitions and reporting. While *I*^2^ values were considerable in most pooled analyses, statistical heterogeneity for prevalence estimates was expected due to the nature of noncomparative proportional data as well as the temporal and geographical variations in study settings.^15^ Random-effects meta-regression did not identify consistent sources of heterogeneity, yet incorporation of additional potential modifiers, such as specific CAR T-cell products, CRS severity, and previous exposure to anthracyclines was precluded by the paucity of data. Furthermore, small-study effects assessment using Doi plots and Luis Furuya-Kanamori indices (eFigures 10-16 in Supplement 1) demonstrated asymmetry for assessed outcomes, suggesting the possibility of publication bias. Considering the a priori expectation that studies describing higher prevalence estimates have a higher likelihood of publication, this may indicate a tendency of currently published data to overestimate the true prevalence of cardiovascular events after CAR T-cell therapy.

## Conclusions

In this proportional meta-analysis exploring the cardiotoxic spectrum of CAR T-cell therapies applied in patients with advanced hematologic malignant neoplasms, a low pooled prevalence of ventricular arrhythmia, myocardial infarction, and cardiovascular mortality over short-term to midterm follow-up was observed. Left ventricular dysfunction and supraventricular arrhythmia were the most commonly reported cardiovascular complications, suggesting that cardiovascular surveillance strategies in CAR T-cell recipients should focus on decreases in ejection fraction and supraventricular arrhythmia. Prospective, multicenter, international registries–based studies systematically applying cardiac testing and recording of cardiac events over a long-term follow-up are needed to confirm these findings and permit documentation of rare cardiovascular complications that may not be captured in the current spectrum of evidence.

## Notes

Supplement 1. **eFigure 1.** PRISMA Flowchart Displaying the Study Selection Process **eFigure 2.** Forest Plot Displaying the Pooled Prevalence of All-Cause Death Using Inverse-Variance Random Effect Models With Freeman-Tukey Double Arcsine Transformation **eFigure 3.** Forest Plot Displaying the Pooled Prevalence of Ventricular Arrhythmia Using a Generalized Linear Mixed Model With Logit Transformation **eFigure 4.** Forest Plot Displaying the Pooled Prevalence of Supraventricular Arrhythmia Using a Generalized Linear Mixed Model With Logit Transformation **eFigure 5.** Forest Plot Displaying the Pooled Prevalence of Reduction in LVEF Using a Generalized Linear Mixed Model With Logit Transformation **eFigure 6.** Forest Plot Displaying the Pooled Prevalence of Heart Failure Events Using a Generalized Linear Mixed Model With Logit Transformation **eFigure 7.** Forest Plot Displaying the Pooled Prevalence of Myocardial Infarction Using a Generalized Linear Mixed Model With Logit Transformation **eFigure 8.** Forest Plot Displaying the Pooled Prevalence of Cardiovascular Death Using a Generalized Linear Mixed Model With Logit Transformation **eFigure 9.** Forest Plot Displaying the Pooled Prevalence of All-Cause Death Using a Generalized Linear Mixed Model With Logit Transformation **eFigure 10.** Doi Plot and Its Associated Luis Furuya-Kanamori Index for the Prevalence of Ventricular Arrhythmia **eFigure 11.** Doi Plot and Its Associated Luis Furuya-Kanamori Index for the Prevalence of Supraventricular Arrhythmia **eFigure 12.** Doi Plot and Its Associated Luis Furuya-Kanamori Index for the Prevalence of Reduction in LVEF **eFigure 13.** Doi Plot and Its Associated Luis Furuya-Kanamori Index for the Prevalence of Heart-Failure Events **eFigure 14.** Doi Plot and Its Associated Luis Furuya-Kanamori Index for the Prevalence of Myocardial Infarction **eFigure 15.** Doi Plot and Its Associated Luis Furuya-Kanamori Index for the Prevalence of Cardiovascular Mortality **eFigure 16.** Doi Plot and Its Associated Luis Furuya-Kanamori Index for the Prevalence of All-Cause Mortality **eTable 1.** Methodological Characteristics of Included Studies **eTable 2.** Results of Random-Effects Meta-Regression Using Patient Age and Proportion of Patients With Lymphoma as Potential Modifiers of Prevalence Estimates **eAppendix 1.** Search Strategy **eAppendix 2.** Quality Assessment of Included Studies Using the JBI Critical Appraisal Checklist for Studies Reporting Prevalence Data

Supplement 2.
Data Sharing Statement
