# Supplementary material for: Cardiovascular Events After Chimeric Antigen Receptor T-Cell Therapy for Advanced Hematologic Malignant Neoplasms: A Meta-Analysis
Source: JAMA Netw Open. 2024 Oct 7;7(10):e2437222. doi: 10.1001/jamanetworkopen.2024.37222 (PMC11459246; doi:10.1001/jamanetworkopen.2024.37222)
Supplement: Supplement 1. — eFigure 1. PRISMA Flowchart Displaying the Study Selection Process eFigure 2. Forest Plot Displaying the Pooled Prevalence of All-Cause Death Using Inverse-Variance Random Effect Models With Freeman-Tukey Double Arcsine Transformation eFigure 3. Forest Plot Displaying the Pooled Prevalence of Ventricular Arrhythmia Using a Generalized Linear Mixed Model With Logit Transformation eFigure 4. Forest Plot Displaying the Pooled Prevalence of Supraventricular Arrhythmia Using a Generalized Linear Mixed Model With Logit Transformation eFigure 5. Forest Plot Displaying the Pooled Prevalence of Reduction in LVEF Using a Generalized Linear Mixed Model With Logit Transformation eFigure 6. Forest Plot Displaying the Pooled Prevalence of Heart Failure Events Using a Generalized Linear Mixed Model With Logit Transformation eFigure 7. Forest Plot Displaying the Pooled Prevalence of Myocardial Infarction Using a Generalized Linear Mixed Model With Logit Transformation eFigure 8. Forest Plot Displaying the Pooled Prevalence of Cardiovascular Death Using a Generalized Linear Mixed Model With Logit Transformation eFigure 9. Forest Plot Displaying the Pooled Prevalence of All-Cause Death Using a Generalized Linear Mixed Model With Logit Transformation eFigure 10. Doi Plot and Its Associated Luis Furuya-Kanamori Index for the Prevalence of Ventricular Arrhythmia eFigure 11. Doi Plot and Its Associated Luis Furuya-Kanamori Index for the Prevalence of Supraventricular Arrhythmia eFigure 12. Doi Plot and Its Associated Luis Furuya-Kanamori Index for the Prevalence of Reduction in LVEF eFigure 13. Doi Plot and Its Associated Luis Furuya-Kanamori Index for the Prevalence of Heart-Failure Events eFigure 14. Doi Plot and Its Associated Luis Furuya-Kanamori Index for the Prevalence of Myocardial Infarction eFigure 15. Doi Plot and Its Associated Luis Furuya-Kanamori Index for the Prevalence of Cardiovascular Mortality eFigure 16. Doi Plot and Its Associated Luis Furuya-Kanamori Index for the Pr [file jamanetwopen-e2437222-s001.pdf]

Supplemental Online Content

Koeckerling D, Reddy RK, Barker J, et al. Cardiovascular events after chimeric antigen receptor T-cell therapy for advanced hematologic malignant neoplasms: a meta-analysis. *JAMA Netw Open*. 2024;7(10):e2437222.
doi:10.1001/jamanetworkopen.2024.37222

eFigure 1. PRISMA Flowchart Displaying the Study Selection Process

eFigure 2. Forest Plot Displaying the Pooled Prevalence of All-Cause Death Using Inverse-Variance Random Effect Models With Freeman-Tukey Double Arcsine Transformation

eFigure 3. Forest Plot Displaying the Pooled Prevalence of Ventricular Arrhythmia Using a Generalized Linear Mixed Model With Logit Transformation

eFigure 4. Forest Plot Displaying the Pooled Prevalence of Supraventricular Arrhythmia Using a Generalized Linear Mixed Model With Logit Transformation

eFigure 5. Forest Plot Displaying the Pooled Prevalence of Reduction in LVEF Using a Generalized Linear Mixed Model With Logit Transformation

eFigure 6. Forest Plot Displaying the Pooled Prevalence of Heart Failure Events Using a Generalized Linear Mixed Model With Logit Transformation

eFigure 7. Forest Plot Displaying the Pooled Prevalence of Myocardial Infarction Using a Generalized Linear Mixed Model With Logit Transformation

eFigure 8. Forest Plot Displaying the Pooled Prevalence of Cardiovascular Death Using a Generalized Linear Mixed Model With Logit Transformation

eFigure 9. Forest Plot Displaying the Pooled Prevalence of All-Cause Death Using a Generalized Linear Mixed Model With Logit Transformation

eFigure 10. Doi Plot and Its Associated Luis Furuya-Kanamori Index for the Prevalence of Ventricular Arrhythmia

© 2024 Koeckerling D et al. *JAMA Network Open*

eFigure 11. Doi Plot and Its Associated Luis Furuya-Kanamori Index for the Prevalence of Supraventricular Arrhythmia

eFigure 12. Doi Plot and Its Associated Luis Furuya-Kanamori Index for the Prevalence of Reduction in LVEF

eFigure 13. Doi Plot and Its Associated Luis Furuya-Kanamori Index for the Prevalence of Heart-Failure Events

eFigure 14. Doi Plot and Its Associated Luis Furuya-Kanamori Index for the Prevalence of Myocardial Infarction

eFigure 15. Doi Plot and Its Associated Luis Furuya-Kanamori Index for the Prevalence of Cardiovascular Mortality

eFigure 16. Doi Plot and Its Associated Luis Furuya-Kanamori Index for the Prevalence of All-Cause Mortality

eTable 1. Methodological Characteristics of Included Studies

eTable 2. Results of Random-Effects Meta-Regression Using Patient Age and Proportion of Patients With Lymphoma as Potential Modifiers of Prevalence Estimates

eAppendix 1. Search Strategy

eAppendix 2. Quality Assessment of Included Studies Using the JBI Critical Appraisal Checklist for Studies Reporting Prevalence Data

This supplemental material has been provided by the authors to give readers additional information about their work.

eFigure 1: PRISMA flowchart displaying the study selection process

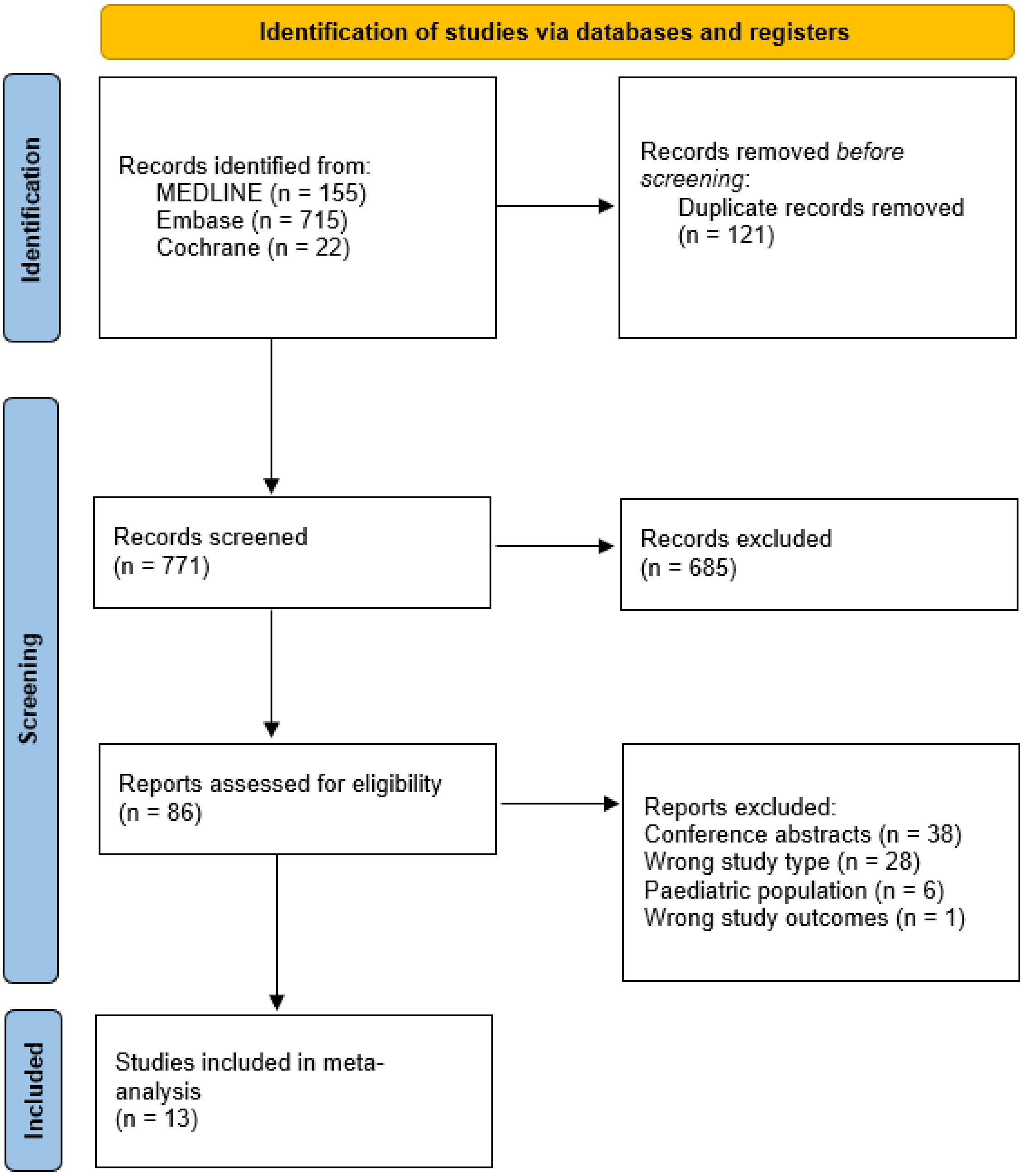

© 2024 Koeckerling D et al. *JAMA Network Open*

eFigure 2: Forest plot displaying the pooled prevalence of all-cause death using inverse-variance random effect models with Freeman-Tukey double arcsine transformation. Error bars represent 95% CIs. Diamonds indicate pooled estimate.

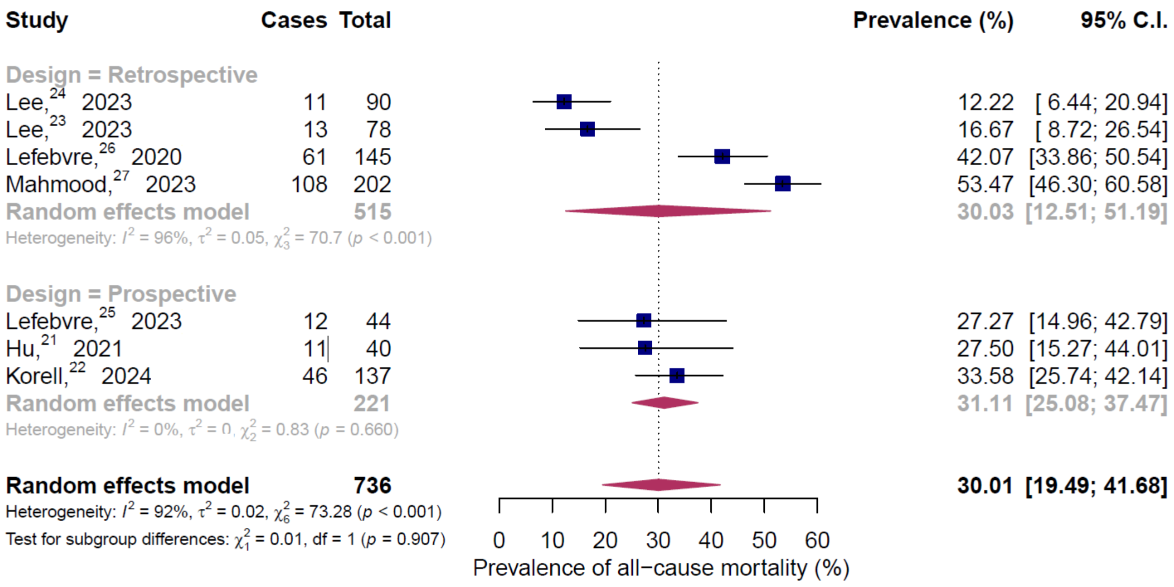

© 2024 Koeckerling D et al. *JAMA Network Open*

eFigure 3: Forest plot displaying the pooled prevalence of ventricular arrhythmia using a generalized linear mixed model with logit transformation. Error bars represent 95% CIs. Diamonds indicate pooled estimate.

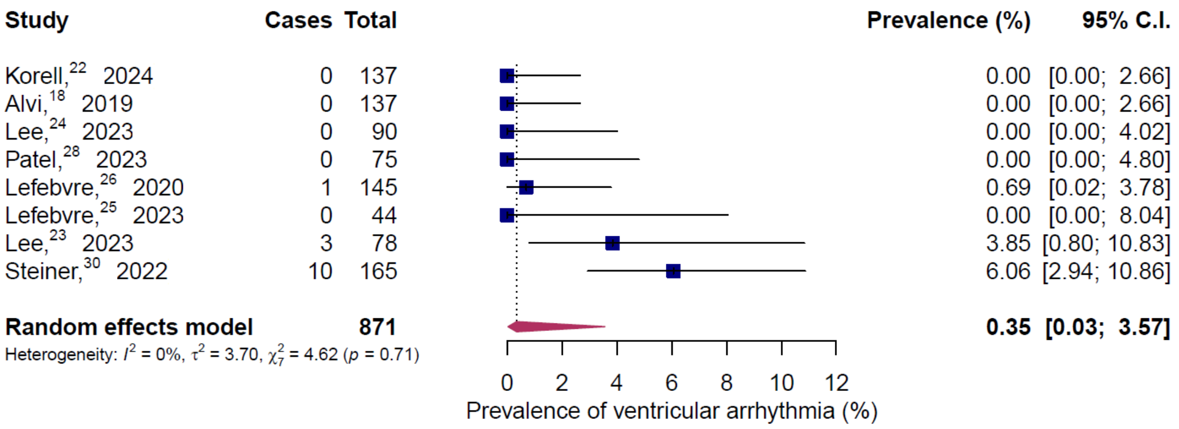

© 2024 Koeckerling D et al. *JAMA Network Open*

eFigure 4: Forest plot displaying the pooled prevalence of supraventricular arrhythmia using a generalized linear mixed model with logit transformation. Error bars represent 95% CIs. Diamonds indicate pooled estimate.

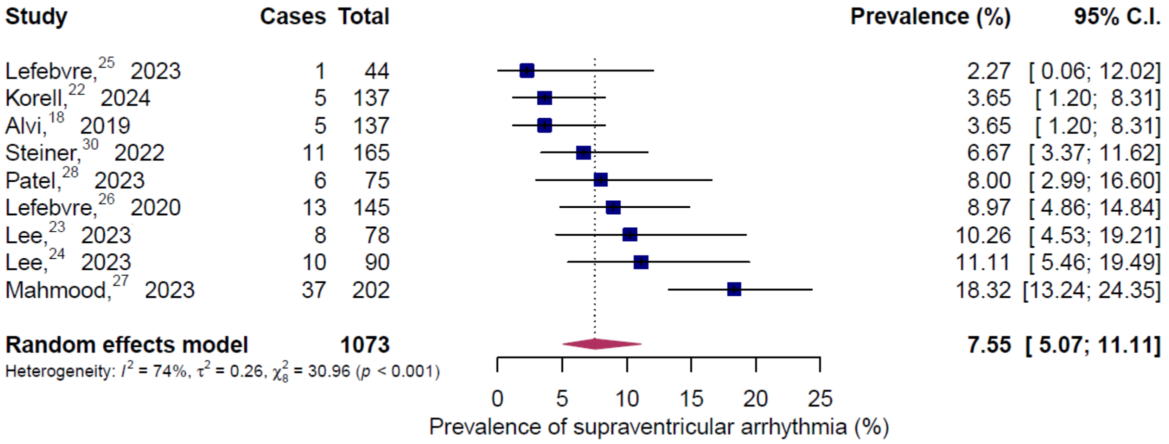

© 2024 Koeckerling D et al. *JAMA Network Open*

eFigure 5: Forest plot displaying the pooled prevalence of reduction in LVEF using a generalized linear mixed model with logit transformation. Error bars represent 95% CIs. Diamonds indicate pooled estimate.

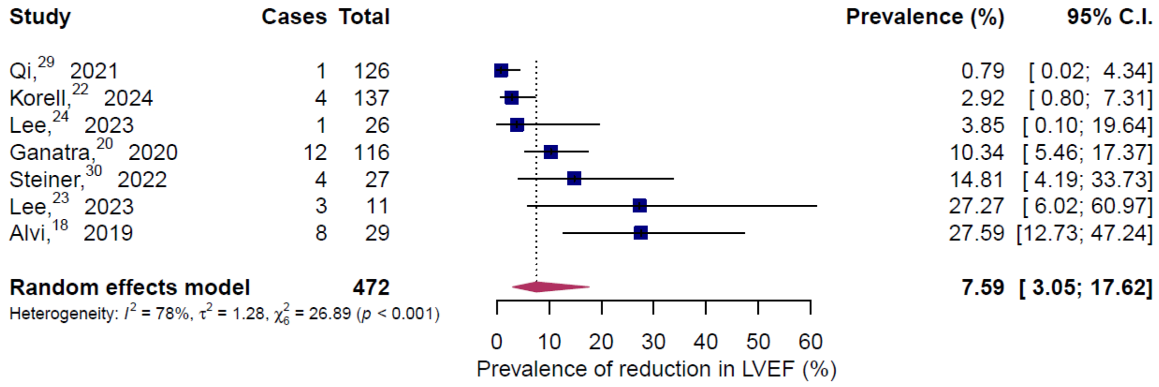

© 2024 Koeckerling D et al. *JAMA Network Open*

eFigure 6: Forest plot displaying the pooled prevalence of heart failure events using a generalized linear mixed model with logit transformation. Error bars represent 95% CIs. Diamonds indicate pooled estimate.

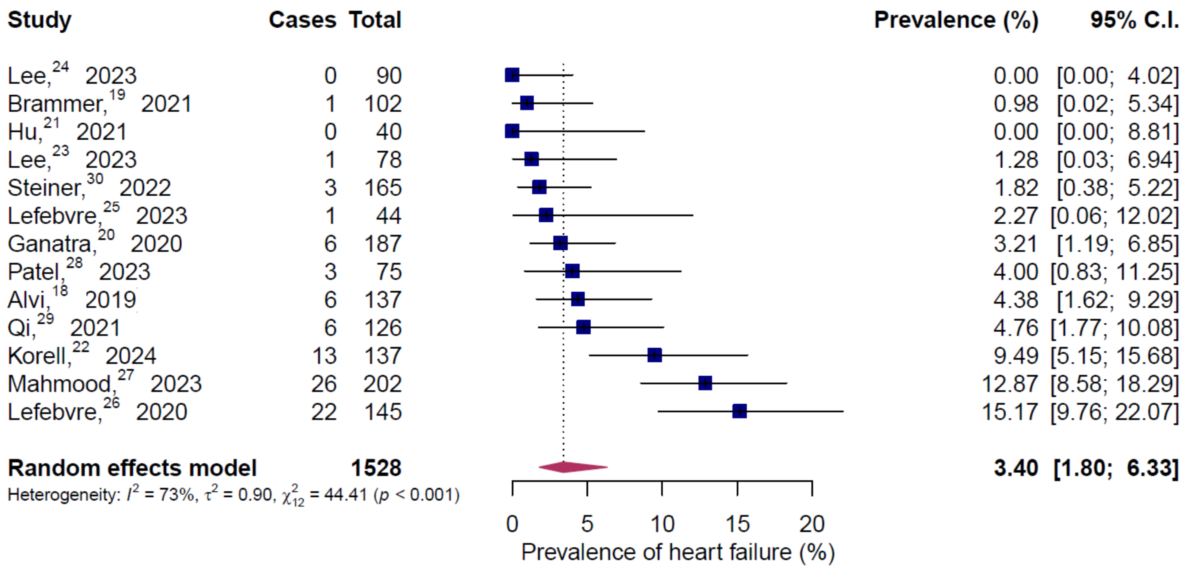

© 2024 Koeckerling D et al. *JAMA Network Open*

eFigure 7: Forest plot displaying the pooled prevalence of myocardial infarction using a generalized linear mixed model with logit transformation. Error bars represent 95% CIs. Diamonds indicate pooled estimate.

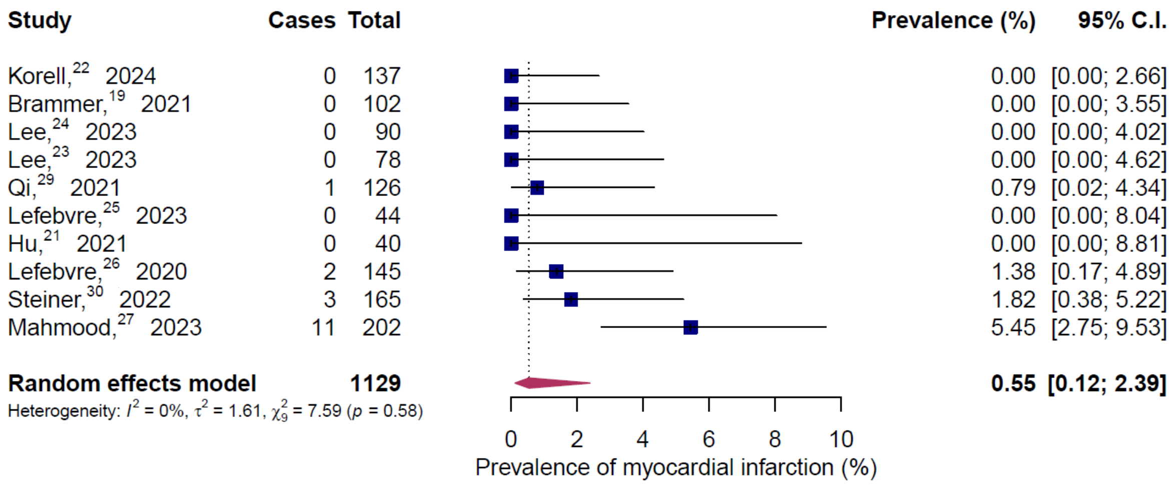

© 2024 Koeckerling D et al. *JAMA Network Open*

eFigure 8: Forest plot displaying the pooled prevalence of cardiovascular death using a generalized linear mixed model with logit transformation. Error bars represent 95% CIs. Diamonds indicate pooled estimate.

Study	Cases	Total	Prevalence (%)	95% C.I.
Korell,[22] 2024	0	137	0.00	[0.00; 2.66]
Brammer,[19] 2021	0	102	0.00	[0.00; 3.55]
Mahmood,[27] 2023	1	202	0.50	[0.01; 2.73]
Lee,[24] 2023	0	90	0.00	[0.00; 4.02]
Steiner,[30] 2022	1	165	0.61	[0.02; 3.33]
Lefebvre,[25] 2023	0	44	0.00	[0.00; 8.04]
Hu,[21] 2021	0	40	0.00	[0.00; 8.81]
Lee,[23] 2023	1	78	1.28	[0.03; 6.94]
Patel,[28] 2023	1	75	1.33	[0.03; 7.21]
Lefebvre,[26] 2020	2	145	1.38	[0.17; 4.89]
Qi,[29] 2021	2	126	1.59	[0.19; 5.62]
Alvi,[18] 2019	6	137	4.38	[1.62; 9.29]
**Random effects model**		**1341**	**0.78**	**[0.33; 1.83]**

Heterogeneity: $I^2 = 0\%$, $\tau^2 = 0.54$, $\chi^2_{11} = 7.94$ ($p = 0.72$)

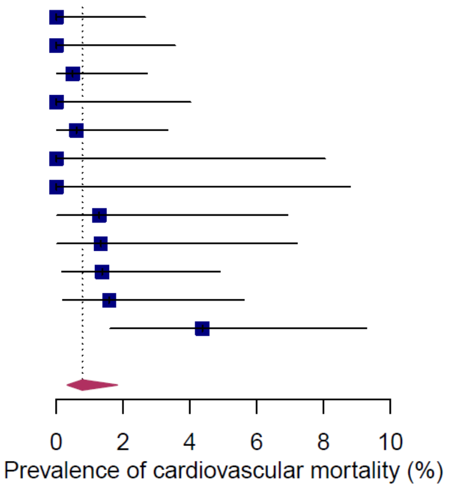

© 2024 Koeckerling D et al. *JAMA Network Open*

eFigure 9: Forest plot displaying the pooled prevalence of all-cause death using a generalized linear mixed model with logit transformation. Error bars represent 95% CIs. Diamonds indicate pooled estimate.

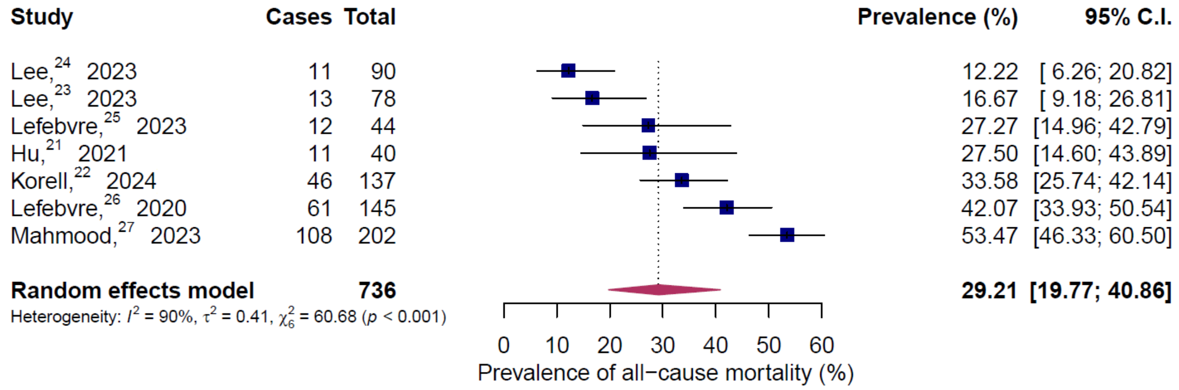

© 2024 Koeckerling D et al. *JAMA Network Open*

eFigure 10: Doi plot and its associated Luis Furuya-Kanamori index for the prevalence ventricular arrhythmia

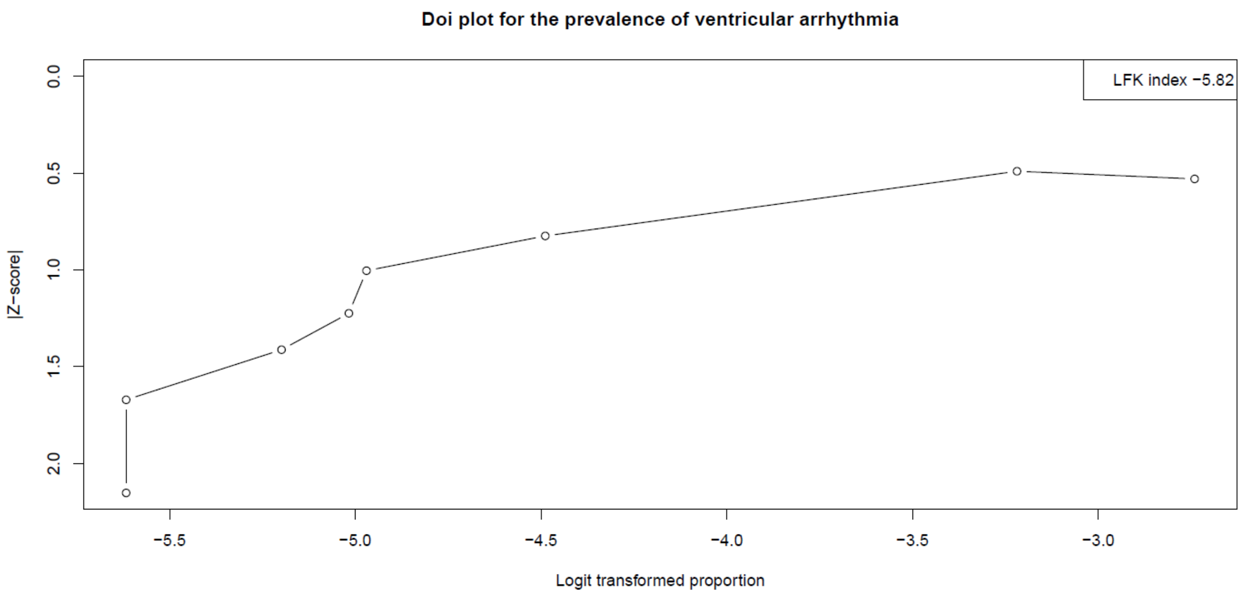

© 2024 Koeckerling D et al. *JAMA Network Open*

eFigure 11: Doi plot and its associated Luis Furuya-Kanamori index for the prevalence of supraventricular arrhythmia

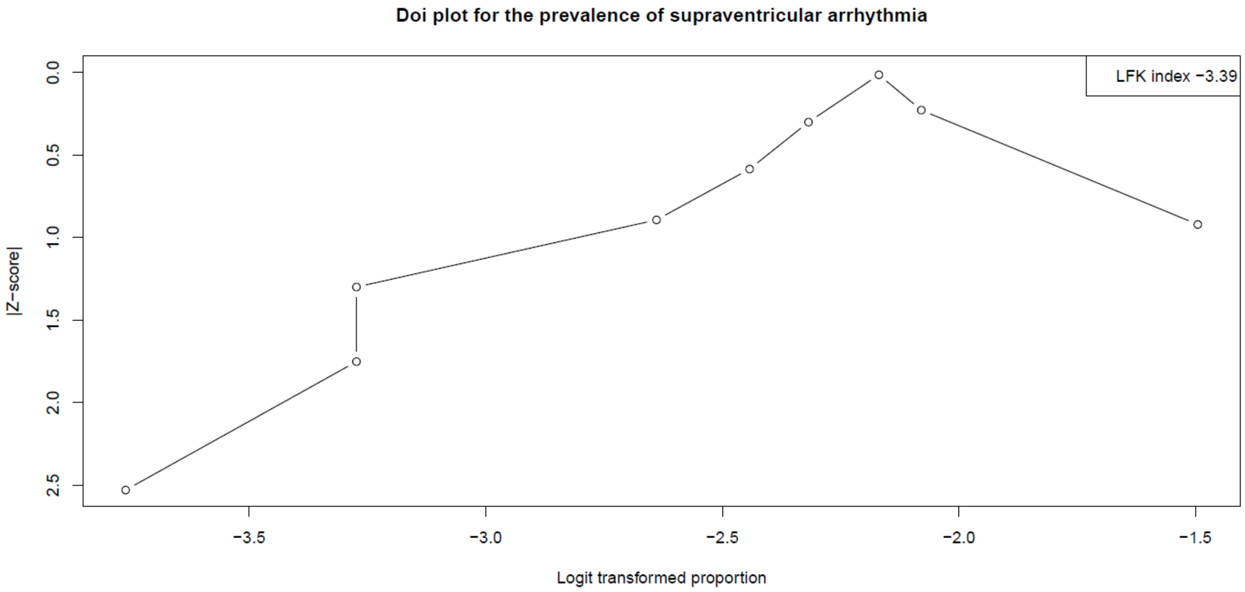

© 2024 Koeckerling D et al. *JAMA Network Open*

eFigure 12: Doi plot and its associated Luis Furuya-Kanamori index for the prevalence of reduction in LVEF

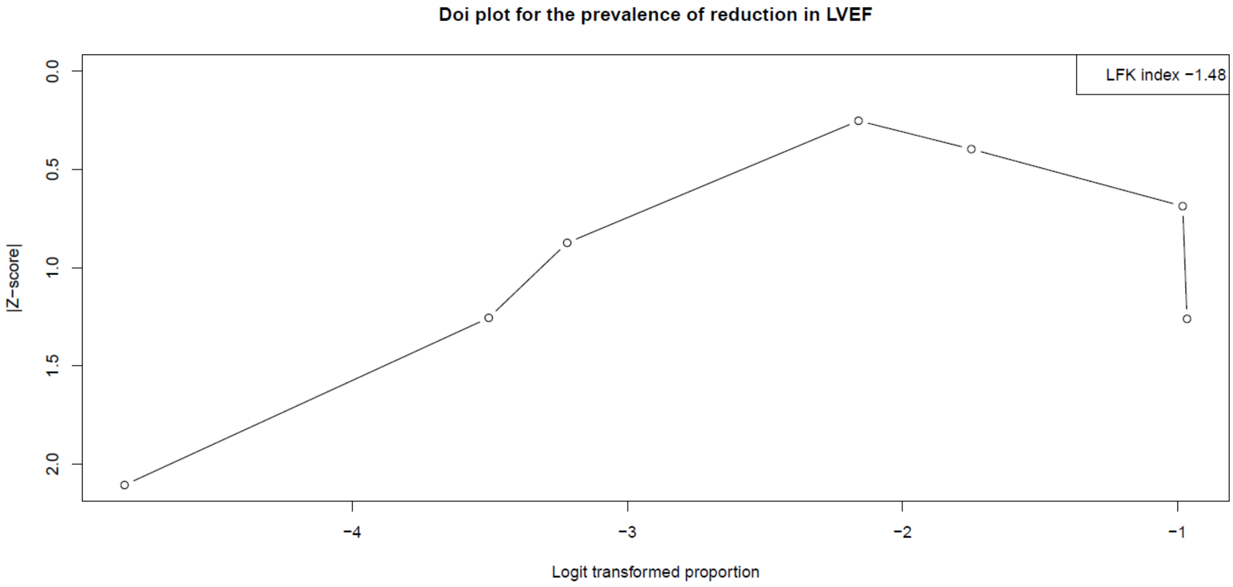

© 2024 Koeckerling D et al. *JAMA Network Open*

eFigure 13: Doi plot and its associated Luis Furuya-Kanamori index for the prevalence of heart-failure events

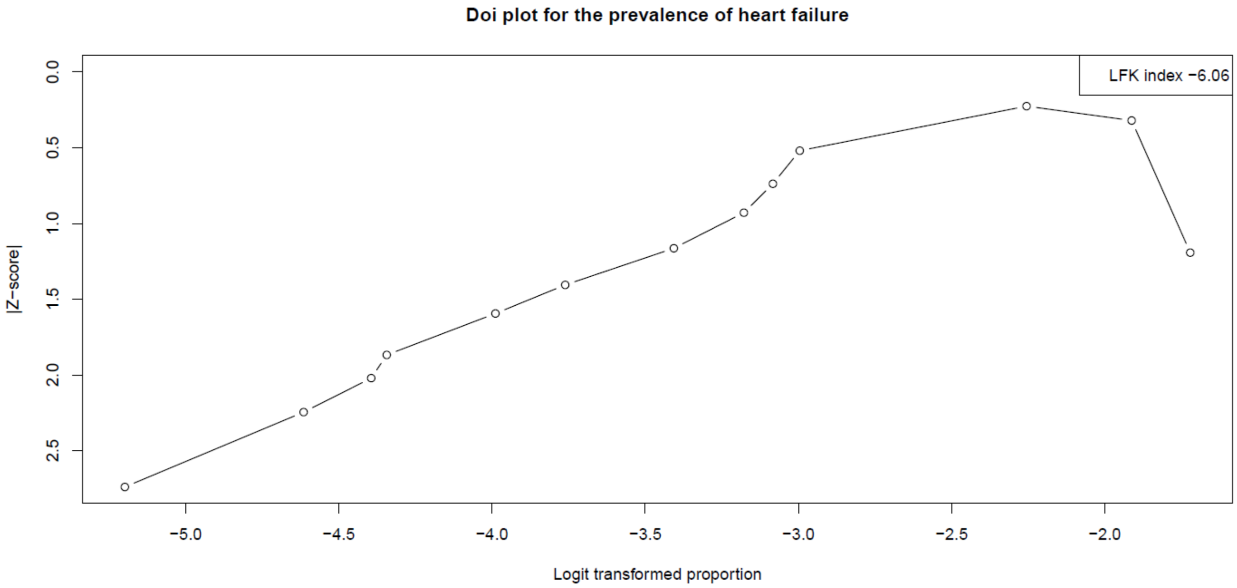

© 2024 Koeckerling D et al. *JAMA Network Open*

eFigure 14: Doi plot and its associated Luis Furuya-Kanamori index for the prevalence of myocardial infarction

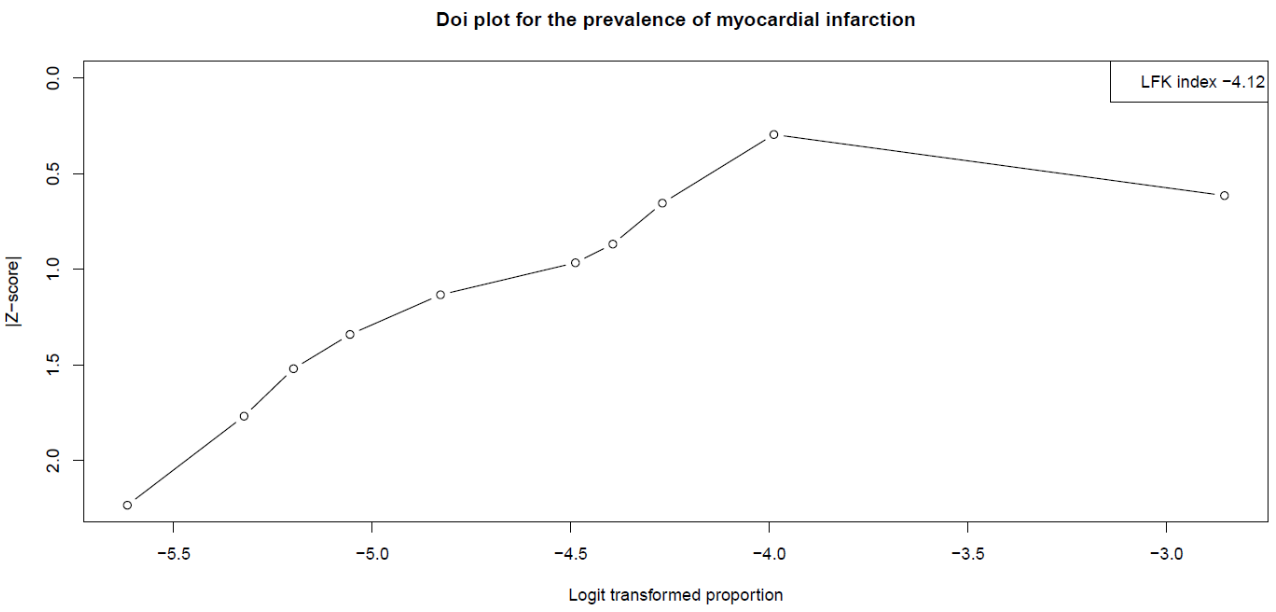

eFigure 15: Doi plot and its associated Luis Furuya-Kanamori index for the prevalence of cardiovascular mortality

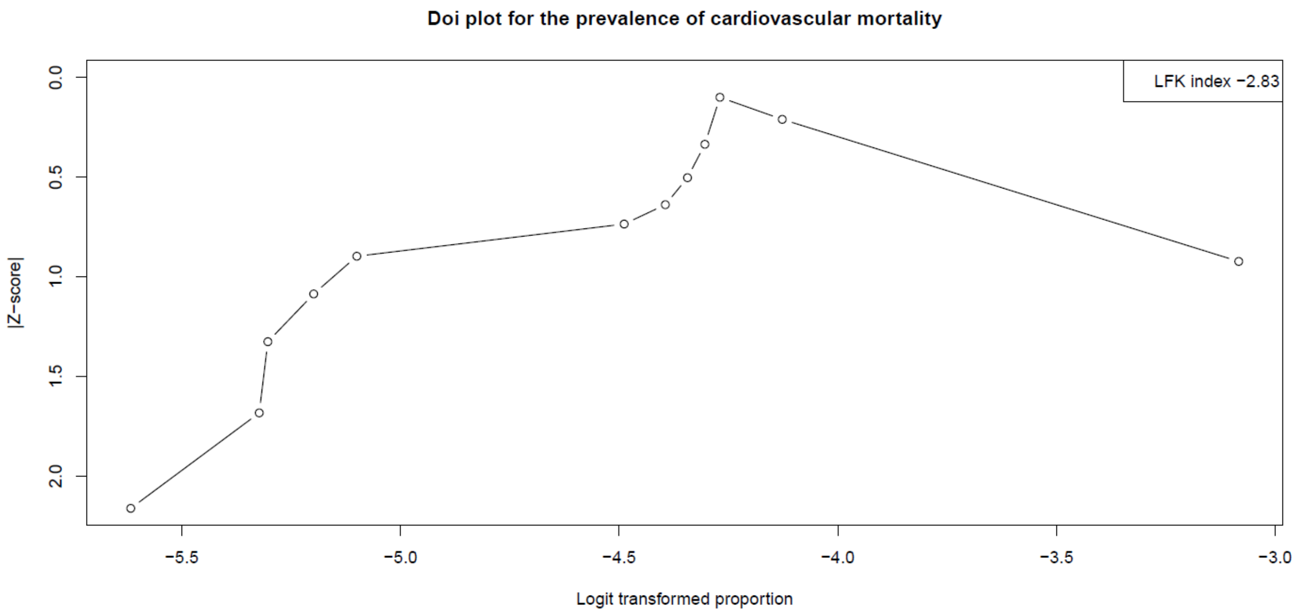

© 2024 Koeckerling D et al. *JAMA Network Open*

eFigure 16: Doi plot and its associated Luis Furuya-Kanamori index for the prevalence of all-cause mortality

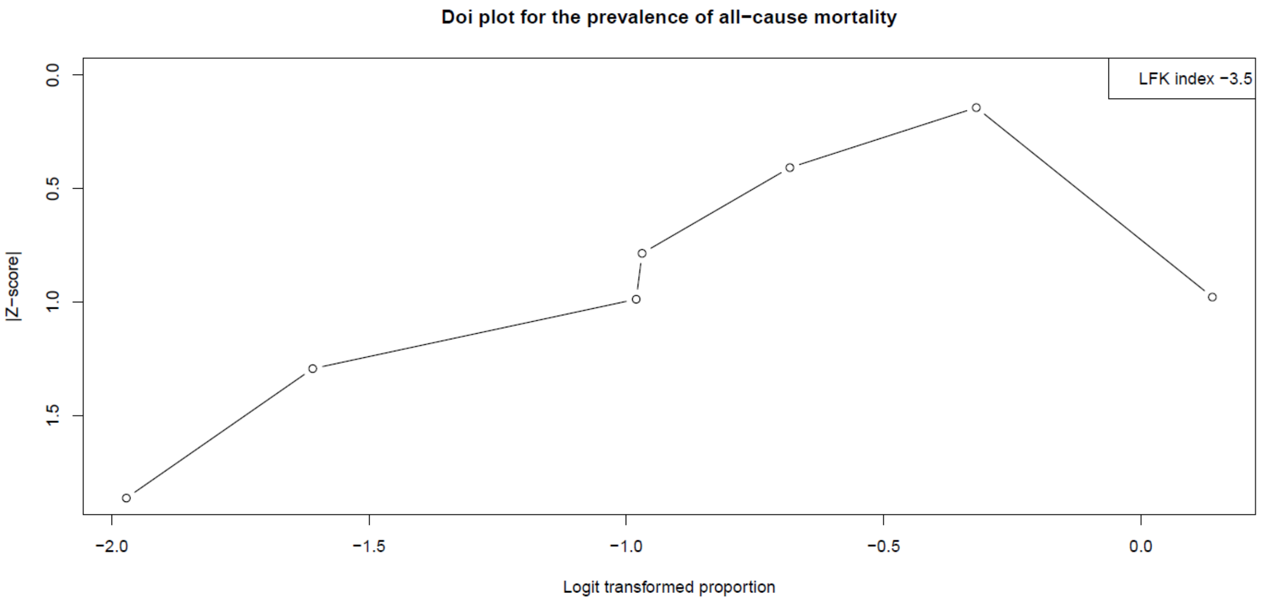

© 2024 Koeckerling D et al. *JAMA Network Open*

eTable 1: Methodological characteristics of included studies

Author, year	Study size	Study design	Centers	Inclusion criteria	CAR T-cell products	Assessment of cardiac events	FU duration
Alvi,[18] 2019	137	Retrosp.	2	All patients receiving CAR T-cell therapy at the 2 study centers between Jan 2016 and Nov 2018	Axicabtagene ciloleucel; Tisagenlecleucel; investigational CAR-T	Cardiac testing was not prespecified and performed at the discretion of the treating physicians.<br>Outcomes were assessed and adjudicated by review of electronic health records by the study team blinded to other variables.	294 ± 205 days
Brammer,[19] 2021	102	Retrosp.	1	Consecutive adult patients receiving CAR T-cell therapy for relapsed/refractory diffuse large B-cell Lymphoma, follicular lymphoma or mantle-cell lymphoma from Jan 2016 to Dec 2019	Axicabtagene ciloleucel; Tisagenlecleucel; Brexucabtagene autoleucel	Cardiac testing was not prespecified and performed at the discretion of the treating physicians. All patient charts were manually searched for adverse events including CRS, neurotoxicity and cardiotoxicity, as well as cardiovascular and all-cause death.	530 ± 499 days
Ganatra,[20] 2020	187	Retrosp.	2	All patients receiving CAR T-cell therapy for refractory or relapsed non-Hodgkin lymphoma at the study centers from Feb 2016 to Apr 2019	Axicabtagene ciloleucel; Tisagenlecleucel	All patients underwent baseline echocardiography. As per institutional protocols follow-up echocardiograms were performed in patients with high-grade CRS (≥grade 2); other patients had follow-up echocardiograms at the discretion of the treating physicians. In-hospital cardiac complications were	168 ± 78 days

						assessed through review of the electronic health records.	
Hu, [21] 2021	40	Prosp.	1	All adult patients receiving CAR T-cell therapy for relapsed/refractory B-cell lymphoma, leukemia or multiple myeloma from Feb 2016 to Oct 2020	Axicabtagene-ciloleucel; Brexucabtagene autoleucel; Tisagenlecleucel	High-sensitivity troponin T and NTproBNP were assessed at baseline, day 1, day 7 and day 21 after CAR T-cell infusion; other cardiac testing was not pre-specified and performed at the discretion of treating physicians. Cardiovascular outcomes were assessed and adjudicated by two physicians through review of electronic health records.	614 ± 304 days
Korell,[22] 2024	137	Prosp.	1	Consecutive patients receiving CAR T-cell therapy at the study center from Oct 2018 to Sep 2022	Axicabtagene ciloleucel; Tisagenlecleucel; Brexucabtagene autoleucel; Idecabtagene vicleucel	Echocardiography was performed at baseline, day 7, and between day 28 and day 180; 12-lead ECGs were performed at baseline, day 7, day 14, day 28, day 56, day 90, day 180. High-sensitivity troponin T and NTproBNP were assessed at baseline, day 1, day 3, day 5, day 7, day 14, day 28, day 56, day 90, day 180.	276 ± 262 days
Lee,[24] 2023	90	Retrosp.	1	Patients with diffuse large B cell lymphoma, mantle cell lymphoma, follicular lymphoma, B-cell acute lymphoblastic leukemia treated with CAR T-cell therapy	Axicabtagene ciloleucel; Tisagenlecleucel; Brexucabtagene autoleucel; Lisocabtagene maraleucel	All patients underwent baseline ECG, transthoracic echocardiogram and troponin I and BNP measurement. Follow-up ECGs, troponin I and BNP levels were performed on day 5 post CAR T-cell infusion and in the event of CRS Grade ≥2. Cardio-oncologists	NA

© 2024 Koeckerling D et al. *JAMA Network Open*

				from Oct 2020 to Oct 2021		reviewed all electrocardiograms and transthoracic echocardiograms. In case of abnormal baseline ECG or troponin/BNP, repeat echocardiogram and cardiac MRI were performed at the discretion of the cardio-oncology team. Two cardiology fellows reviewed patients' clinical progress ≥3 times a week; in case of cardiac events, these events were adjudicated and managed by the cardio-oncology attending. After index hospitalisation, patients were seen in cardio-oncology outpatient clinic 2 times per week until day 30, then at 3,6, and 12 months.	
Lee,[23] 2023	78	Retrosp.	1	All consecutive patients with relapsed and refractory multiple myeloma undergoing CAR T-cell therapy with Idecabtagene vicleucel from May 2021 to Oct 2022.	Idecabtagene vicleucel	All patients received baseline cardiac workup including ECG, transthoracic echocardiogram, troponin I and BNP measurements. Repeat echocardiograms were performed at the discretion of treating physicians. All cardiac outcomes were assessed and adjudicated by cardio-oncologists through review of electronic medical records.	NA
Lefebvre,[25] 2023	44	Prosp.	1	All consecutive adult patients with CD19 malignancies treated with commercial CAR	NA	All patients underwent an echocardiogram and cardiac biomarker measurement at baseline, at 2 days, 1 week, 1 month and 6 months post CAR	487 ± 264 days

© 2024 Koeckerling D et al. *JAMA Network Open*

				T-cell products from Jul 2019 to Feb 2022		T-cell infusion. In the event of CRS, a repeat echocardiogram was performed within 72h. Electronic health records of all patients were reviewed at 1 year post CAR T-cell infusion for further cardiac events. All cardiac events were adjudicated by 2 cardiologists blinded to all other clinical and echocardiographic information.	
Lefebvre,[26] 2020	145	Retrosp.	1	All consecutive adult patients with CD19 malignancy treated with CAR T-cell products from Aug 2010 and Jan 2019	NA	Cardiac events were assessed and adjudicated through review of electronic health records by two independent cardiologists blinded to all other clinical and echocardiographic parameters.	599 ± 813 days
Mahmood,[27] 2023	202	Retrosp.	4	Consecutive adult patients receiving CAR T-cell products for CD19 malignancies at the study centers from Feb 2010 to Feb 2021	Axicabtagene ciloleucel; Tisagenlecleucel; Lisocabtagene maraleucel	Cardiac biomarkers were collected before and after CAR T-cell infusion based on the institutional protocols. Cardiac testing was not pre-specified and performed at the discretion of treating physicians. Cardiac events were assessed and adjudicated through manual review of electronic health records	349 ± 405 days
Patel,[28] 2023	75	Retrosp.	1	Patients with an available baseline echocardiogram within 6 months prior to	NA	Cardiac testing was not prespecified and performed at the discretion of the treating physicians. Cardiac events were assessed and adjudicated through	NA

© 2024 Koeckerling D et al. *JAMA Network Open*

				receiving CAR T-cell therapy at the study center from 2016 to 2020		manual review of electronic health records.	
Qi,[29] 2021	126	Retrosp.	1	Patients receiving CAR T-cell products at the study center from Jan 2019 to Nov 2020	NA	Cardiac biomarkers including high sensitivity troponin T and NTproBNP were collected at baseline, day 1-3, day 4-6, day 7-10, day 11-13, day 14-16, day 17-20, day 21-24, day 25-30, day 31-40, day 41-50. Conduction of echocardiography and electrocardiograms was not pre-specified and left to the discretion of treating physicians. Cardiac events were assessed and adjudicated through manual review of electronic health records.	NA
Steiner,[30] 2022	165	Retrosp.	1	Consecutive adult patients with relapsed or refractory aggressive large B-cell lymphoma treated with CAR T-cell products from Jan 2018 to Apr 2020.	Axicabtagene ciloleucel; Tisagenlecleucel	Cardiac testing was not prespecified and performed at the discretion of the treating physicians. Cardiac events were assessed and adjudicated through manual review of electronic health records. All ECGs and echocardiograms were reviewed by a cardiologist.	493 ± 24 days

CRS – cytokine release syndrome; ECG – electrocardiogram; NTproBNP – N-terminal pro brain natriuretic peptide;

© 2024 Koeckerling D et al. *JAMA Network Open*

eTable 2: Results of random-effects meta-regression using patient age and proportion of patients with lymphoma as potential modifiers of prevalence estimates

**Outcome**	**Modifier**	**P-value**
Heart-failure events	Age	0.01
	Proportion of lymphoma	0.29
Myocardial infarction	Age	0.10
	Proportion of lymphoma	0.92
Cardiovascular mortality	Age	0.82
	Proportion of lymphoma	0.22

© 2024 Koeckerling D et al. *JAMA Network Open*

eAppendix 1: Search Strategy

**Database**	**Provider**	**No. of results**
MEDLINE	Ovid	155
Embase	Ovid	715
Cochrane CENTRAL	Wiley	22

All databases searched from their inception to the date shown (searches performed on Monday 26th February 2024), no language or date limits applied.

Ovid MEDLINE(R) ALL <1946 to February 23, 2024>

1 (t-cell* adj1 therap*).mp. or (tcell* adj1 therap*).tw. [mp=title, book title, abstract, original title, name of substance word, subject heading word, floating sub-heading word, keyword heading word, organism supplementary concept word, protocol supplementary concept word, rare disease supplementary concept word, unique identifier, synonyms, population supplementary concept word, anatomy supplementary concept word] 8837
2 ((chimeric* adj3 antigen receptor*) or (chimeric* adj3 immunoreceptor*) or (chimeric* adj3 T cell receptor*) or (chimeric* adj3 Tcell receptor*)).tw,kf. 11013
3 Receptors, Chimeric Antigen/4875
4 ((artificial* adj3 T cell receptor*) or (artificial* adj3 Tcell receptor*)).tw,kf. 12
5 axicabtagene*.tw,kf. 441
6 (yescarta* or axi-cel* or KTE-C19 or "CTL 019").tw,kf. 286
7 tisagenlecleucel*.tw,kf. 518
8 (kymriah* or CART-19 or CART19).tw,kf. 213
9 Lisocabtagene*.tw,kf. 107
10 (liso-cel* or JCAR017*).tw,kf. 53
11 Brexucabtagene*.tw,kf. 81
12 Idecabtagene vicleucel.tw,kf. 105
13 Abecma.tw,kf. 6
14 or/1-1315223
15 Cardiotoxicity/ 4757
16 cardiotoxic*.tw,kf. 19197
17 exp heart/ or heart.tw,kf. or cardiac.tw,kf. 1672885
18 (ae or co or mo or po or to).fs. 4994874
19 17 and 18 449502
20 exp Cardiovascular Diseases/ci [Chemically Induced] 89368
21 Arrhythmias, Cardiac/68172
22 arrhythm*.tw,kf. 121095
23 exp Cardiomyopathies/ 114772
24 cardiomyopath*.tw,kf. 92156
25 exp Heart Failure/ 151997
26 ((cardiac or heart) adj2 fail*).tw,kf. 241303

© 2024 Koeckerling D et al. *JAMA Network Open*

27 ejection fraction.tw,kf. 85218
28 Ventricular Dysfunction, Left/ 31389
29 exp Acute Coronary Syndrome/ 20724
30 acute coronary syndrome.tw,kf. 31297
31 LVEF.mp. or left ventricular ejection fraction.tw,kf. [mp=title, book title, abstract, original title, name of substance word, subject heading word, floating sub-heading word, keyword heading word, organism supplementary concept word, protocol supplementary concept word, rare disease supplementary concept word, unique identifier, synonyms, population supplementary concept word, anatomy supplementary concept word] 41006
32 myocardial infarction/ or myocardial infarction.tw,kf. 280540
33 exp Myocardial Ischemia/ 477715
34 myocardial isch?emi*.tw,kf. 38742
35 exp Troponin/ 20958
36 troponin.tw,kf.33496
37 myocarditis.tw,kf. 21825
38 (cardiovascular adj2 (death or mortality)).tw,kf. 37000
39 exp Myocarditis/ 17551
40 15 or 16 or 19 or 20 or 21 or 22 or 23 or 24 or 25 or 26 or 27 or 28 or 29 or 30 or 31 or 32 or 33 or 34 or 35 or 36 or 37 or 38 or 39 1313418
41 14 and 40 155

Embase <1974 to 2024 February 23>

1 (t-cell* adj1 therap*).mp. or (tcell* adj1 therap*).tw. 17333
2 ((chimeric* adj3 antigen receptor*) or (chimeric* adj3 immunoreceptor*) or (chimeric* adj3 T cell receptor*) or (chimeric* adj3 Tcell receptor*)).tw,kf. 20166
3 chimeric antigen receptor t-cell immunotherapy/ 11633
4 chimeric antigen receptor T-cell/ 12071
5 ((artificial* adj3 T cell receptor*) or (artificial* adj3 Tcell receptor*)).tw,kf. 46
6 axicabtagene*.tw,kf. 1496
7 (yescarta* or axi-cel* or KTE-C19 or "CTL 019").tw,kf. 1566
8 axicabtagene ciloleucel/ 2515
9 tisagenlecleucel T/ 2835
10 tisagenlecleucel*.tw,kf. 1492
11 (kymriah* or CART-19 or CART19).tw,kf. 1005
12 lisocabtagene maraleucel/ 776
13 Lisocabtagene*.tw,kf. 381
14 (liso-cel* or JCAR017*).tw,kf. 308
15 brexucabtagene autoleucel/ 609
16 Brexucabtagene*.tw,kf. 228
17 idecabtagene vicleucel/ 681
18 Abecma.tw,kf. 94
19 or/1-1834023
20 cardiotoxicity/ 54047

21 cardiotoxic*.tw,kf. 28091
22 exp heart/ or heart.tw,kf. or cardiac.tw,kf. 2339152
23 (ae or si or to).fs. 1950930
24 22 and 23 114341
25 chemically induced disorder/ 79411
26 exp cardiovascular disease/ 5137785
27 25 and 26 12515
28 arrhythm*.tw,kf. 184756
29 heart arrhythmia/ 144842
30 cardiomyopath*.tw,kf. 150343
31 cardiomyopathy/ 70887
32 exp heart failure/ 660728
33 ((cardiac or heart) adj2 fail*).tw,kf. 397159
34 exp heart ejection fraction/ 200346
35 heart left ventricle failure/ 32504
36 exp acute coronary syndrome/ 77509
37 acute coronary syndrome.tw,kf. 57616
38 LVEF.mp. or left ventricular ejection fraction.tw,kf. 90221
39 heart infarction/ 317256
40 myocardial infarction.tw,kf. 326609
41 exp heart muscle ischemia/ 102858
42 myocardial isch?emi*.tw,kf. 54363
43 exp troponin/ 89649
44 troponin.tw,kf. 59218
45 exp myocarditis/ 41076
46 myocarditis.tw,kf. 30732
47 (cardiovascular adj2 (death or mortality)).tw,kf. 58752
48 cardiovascular mortality/ 64351
49 20 or 21 or 24 or 27 or 28 or 29 or 30 or 31 or 32 or 33 or 34 or 35 or 36 or 37 or 38 or 39 or 40 or 41 or 42 or 43 or 44 or 45 or 46 or 47 or 48 1661216
50 19 and 49 715

Search Name: CAR-T cell therapy
Date Run: 27/02/2024 01:48:54
Comment: 26.2.24

ID Search Hits
#1 (t-cell* near/1 therap*) or (tcell* near/1 therap*) 330
#2 ((chimeric* near/3 antigen receptor*) or (chimeric* near/3 immunoreceptor*) or (chimeric* near/3 T cell receptor*) or (chimeric* near/3 Tcell receptor*)) 340
#3 [mh "Receptors, Chimeric Antigen"] 24
#4 (artificial* near/3 (T NEXT cell NEXT receptor*)) or (artificial* near/3 (Tcell NEXT receptor*)) 2
#5 axicabtagene* 67

© 2024 Koeckerling D et al. *JAMA Network Open*

#6 yescarta* or axi-cel* or KTE-C19 or "CTL 019" 69
#7 tisagenlecleucel* 32
#8 (kymriah* or CART-19 or CART19) 17
#9 Lisocabtagene* 21
#10 (liso-cel* or JCAR017*) 24
#11 Brexucabtagene* 1
#12 Idecabtagene vicleucel 16
#13 Abecma 0
#14 5-#13 538
#15 [mh Cardiotoxicity] 203
#16 cardiotoxic* 2285
#17 [mh heart] or heart or cardiac 223733
#18 MeSH descriptor: [] explode all trees and with qualifier(s): [adverse effects - AE, complications - CO, mortality - MO, poisoning - PO, toxicity - TO] 268839
#19 #17 and #18 44513
#20 MeSH descriptor: [Cardiovascular Diseases] explode all trees and with qualifier(s): [chemically induced - CI] 4448
#21 MeSH descriptor: [Arrhythmias, Cardiac] explode all trees 13560
#22 arrhythm* 15922
#23 [mh Cardiomyopathies] 2684
#24 cardiomyopath* 5673
#25 [mh "Heart Failure"] 14344
#26 (cardiac or heart) near/2 fail* 39190
#27 ejection fraction 18674
#28 [mh "Ventricular Dysfunction, Left"] 2588
#29 [mh "Acute Coronary Syndrome"] 3078
#30 acute coronary syndrome 8639
#31 LVEF or left ventricular ejection fraction 14088
#32 [mh "myocardial infarction"] 15580
#33 myocardial infarction 37736
#34 [mh "Myocardial Ischemia"] 38574
#35 myocardial isch?emi* 17553
#36 [mh Troponin] or troponin 5343
#37 myocarditis 1387
#38 [mh myocarditis] 139
#39 cardiovascular near/2 (death or mortality) 10310
#40 22-#16, #19-#39 139611
#41 #14 and #40 22

eAppendix 2: Quality assessment of included studies using the JBI critical appraisal checklist for studies reporting prevalence data

Author	Sample frame appropriate?	Study participants sampled appropriately?	Sample size adequate?	Study subjects and setting described in detail?	Data analysis conducted with sufficient coverage of identified sample?	Valid methods for identification of the condition?	Condition measured in a standard way?	Appropriate statistical analysis?	Response rate adequate?	Overall Score
Lefebvre 2023	Yes	Yes	No	Yes	Yes	Yes	Yes	Yes	Yes	8/9
Hu 2021	Yes	Yes	No	Yes	Yes	Yes	No	Yes	Yes	7/9
Korell 2024	Yes	Yes	Yes	Yes	Yes	Yes	Yes	Yes	Yes	9/9
Patel 2023	No	No	Yes	No	Yes	No	No	Yes	Yes	5/9
Alvi 2019	Yes	Yes	Yes	Yes	Yes	Yes	No	Yes	No	7/9
Lefebvre 2020	Yes	Yes	Yes	Yes	Yes	Yes	No	Yes	Yes	8/9
Lee 2023	Yes	Yes	Yes	Yes	Yes	No	Yes	Yes	Yes	8/9
Ganatra 2020	Yes	Yes	Yes	Yes	Yes	No	Yes	Yes	Yes	8/9
Brammer 2021	Yes	Yes	Yes	Yes	No	No	No	No	Yes	5/9
Mahmood 2023	Yes	Yes	Yes	Yes	Yes	No	No	Yes	Yes	7/9
Steiner 2022	Yes	Yes	Yes	Yes	Yes	Yes	No	Yes	No	7/9
Lee 2023	Yes	Yes	No	Yes	Yes	Yes	Yes	Yes	No	7/9
Qi 2021	Yes	Yes	Yes	No	Yes	No	No	No	No	4/9

© 2024 Koeckerling D et al. *JAMA Network Open*

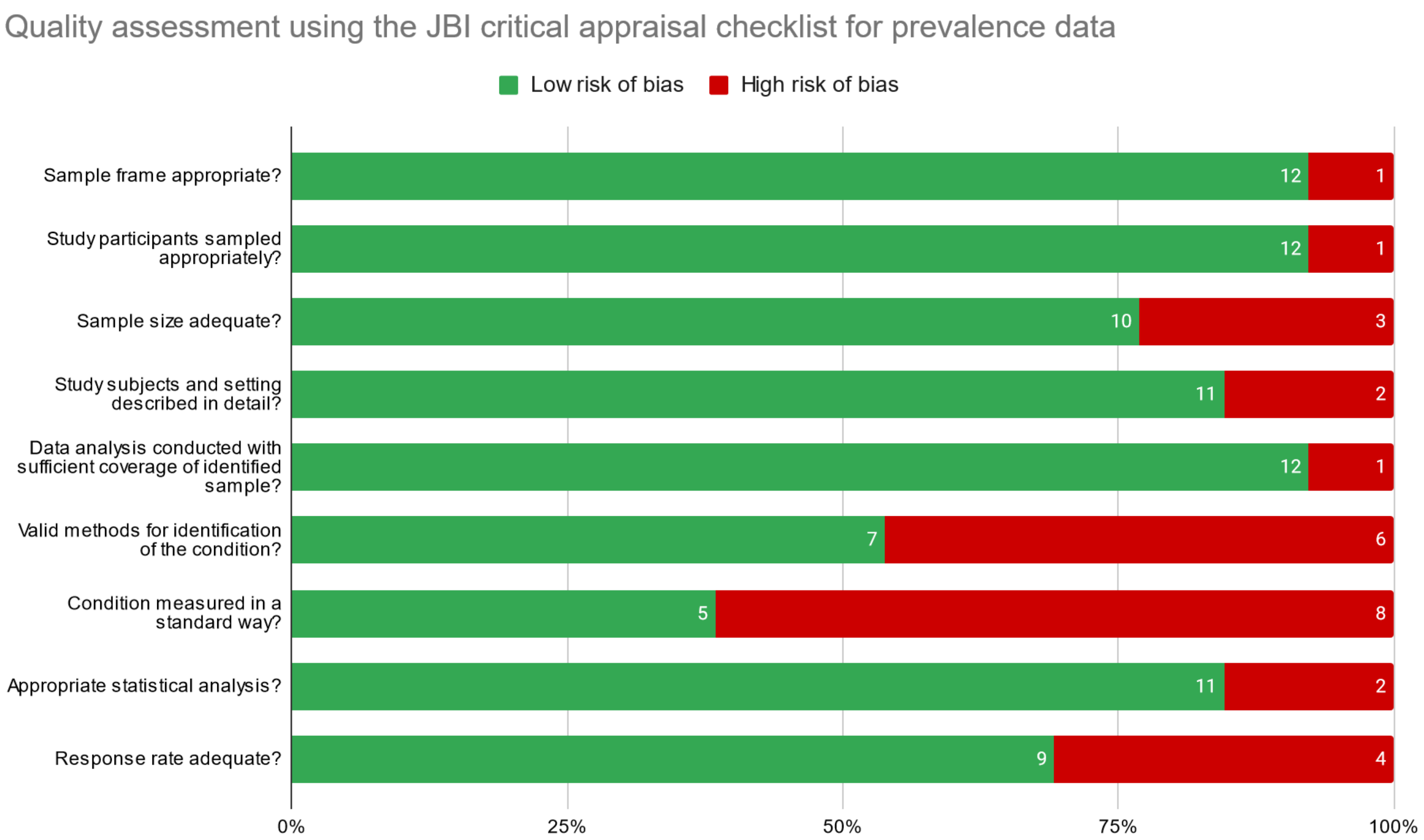

© 2024 Koeckerling D et al. *JAMA Network Open*
