# Supplementary material for: Cardiovascular Events After Chimeric Antigen Receptor T-Cell Therapy for Advanced Hematologic Malignant Neoplasms: A Meta-Analysis
Source: JAMA Netw Open. 2024 Oct 7;7(10):e2437222. doi: 10.1001/jamanetworkopen.2024.37222 (PMC11459246; doi:10.1001/jamanetworkopen.2024.37222)
Supplement: Supplement 2. — Data Sharing Statement [file jamanetwopen-e2437222-s002.pdf]

Data Sharing Statement

Koeckerling. Cardiovascular Events After Chimeric Antigen Receptor T-Cell Therapy for Advanced Hematologic Malignant Neoplasms. *JAMA Netw Open*. Published October 07, 2024. doi:10.1001/jamanetworkopen.2024.37222

Data
Data available: No

Additional Information
Explanation for why data not available: meta-analysis of published data (by us and other groups)
